# Identity of Oxygen-Rich Nickel Oxides as Oxosuperoxides and Oxoperoxides and Their Heterostructures

**DOI:** 10.1021/acs.inorgchem.5c01477

**Published:** 2025-07-25

**Authors:** Radovan Bujdák, Mariana Derzsi, Kamil Tokár

**Affiliations:** † Advanced Technologies Research Institute, Faculty of Materials Science and Technology in Trnava, 61791 Slovak University of Technology in Bratislava, 917 24 Trnava, Slovakia; ‡ Institute of Physics, Slovak Academy of Sciences, 845 11 Bratislava, Slovakia

## Abstract

The chemical identity of oxygen-rich nickel oxides was investigated employing Density Functional Theory calculations performed for Ni-substituted transition-metal pentoxides M_2_O_5_, which have the potential to host M^5+^ species, while the ground-state structure was predicted using evolutionary algorithms for crystal structure prediction. Our results have shown that Ni^5+^ is unlikely to stabilize in the oxide environment and will immediately reduce to more stable nickel oxidation states (Ni^4+^–Ni^2+^) through the formation of molecular oxygen species, while both superoxide and peroxide species are equally likely. The resulting oxoperoxide and oxosuperoxide phases represent oxygen-enriched versions of the already well-known or long-suspected binary nickel oxides (NiO, Ni_2_O_3_, NiO_2_) and their heterostructures including O_2_-decorated rock salt NiO, O-enriched layered CdI_2_-type NiO_2_, a der Waals heterostructure of NiO_2_ and NiO­(O_2_) as well as the monoclinic *C*2/*c* form (high-pressure V_2_O_5_ type structure), which is common to all known pentoxides, and in the case of nickel takes the form of oxosuperoxide Ni_2_O_3_(O_2_). All predicted models, although dynamically stable, were found to be highly unstable in relation to reduction to nickel monoxide, the most stable nickel oxide phase, which provides one explanation for why oxygen-rich nickel oxide phases beyond NiO_2_ have not yet been observed.

## Graphical abstract

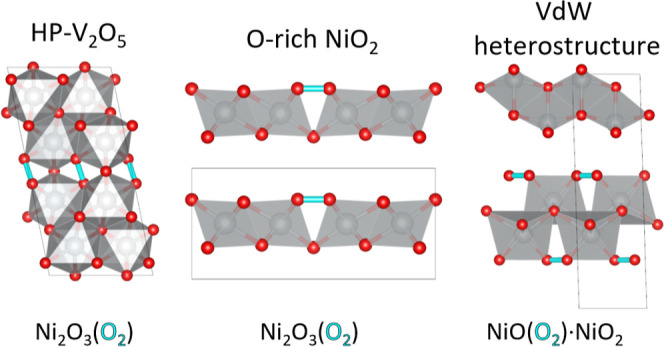

## 1 Introduction

Binary nickel oxides and their derivatives, including various (oxo)­hydroxides and hydrated forms, as well as intercalated and doped phases, show high potential for a broad range of energy-related sensors and environmental remediation applications. The well-known nickel monoxide NiO is a p-type semiconductor with excellent electrical and optical properties, whose mesoporous nanoarchitectures, including thin films, composites, and encapsulated nanoparticles, additionally exhibit superior electrochemical performance. These characteristics make NiO an attractive material for advanced (electrochemical) energy storage, for example, as freestanding electrodes for developing high-performance pseudocapacitors.^1–3^ Nickel monoxide is also becoming attractive as a novel versatile metal oxide-based gas sensor, including formaldehyde, H_2_, and NO_2_ gas sensing.^4–6^ Another nickel oxide phase, NiO_2_ is emerging as an attractive layered cathode component for lithium and novel advanced multivalent metal ionization batteries, in which the Li intercalates are replaced by Mg, Ca, and Zn.^7,8^ The NiO_2_ sheets have also emerged as outstanding catalysts for oxygen evolution reactions in promising solar water splitting technology while outperforming its hydrogenated constituent, NiOOH.^8^ Similar attractive properties have also been claimed for the yet-to-be confirmed Ni_2_O_3_ phase. The three phases of nickel oxide also show promising potential for the photocatalytic capture and degradation of industrial effluents such as methane, wastewater pollutants, congo red dye, and 4-chlorophenol.^9–13^ Furthermore, nickel oxide-based catalysts have proved to have a huge potential in the ethane oxidative dehydrogenation reaction.^14^

Nickel (oxo)­hydroxides, Ni­(OH)_2_, NiOOH, and Ni_2_O_3_H, in turn, have been of huge importance to the commercial alkaline battery industry for over a century. These phases, together with numerous hydrated polymorphs and phase transformations between them, define charge, discharge, and overcharge states, as well as conversion between them and aging mechanisms. These processes involve the evolution of various nickel oxidation states, and some of these processes have been understood only recently.^15–17^

Despite the importance of nickel oxides, nothing is known about the potential formation of oxygen-rich nickel oxides beyond NiO_2_, including nickel peroxides and superoxides, as well as the formation of nickel species beyond Ni^4+^.

The binary phases of nickel oxide mentioned so far in the literature account for NiO, Ni_2_O_3_, and NiO_2_, where the expected oxidation state of nickel is formally Ni^2+^, Ni^3+^, and Ni^4+^, respectively. On the other hand, no inorganic compounds with Ni^5+^ are known, although the oxidation state +5 and the electronic configuration 3d^5^ that correspond to Ni^5+^ are stable for other d elements. The oxidation state 5+ is commonly observed for group 5 elements (V, Nb, and Ta) in binary metal pentoxides M_2_O_5_, with rich structural diversity. Formation of Fe^5+^ was also repeated in a number of oxide perovskites.^18,19^ The electronic configuration 3d^5^ is also commonly observed for manganese and iron in the form of Mn^2+^ or Fe^3+^, respectively, in a number of binary oxide phases.^20–24^ There are also increasing numbers of reports on less common Co^4+^(3d^5^) in oxygen environments.^25–31^ Unusually high oxidation states are often less stable, and the corresponding species have a tendency toward charge disproportionation or reduction. The former is the case of Cr^5+^ in the pentoxide Cr_2_O_5_ for which two disproportionation scenarios are feasible, including 2Cr^5+^ → Cr^4+^Cr^6+^ ^32^ and 6Cr^5+^ → Cr_2_^3+^Cr_4_^6+^.^33^ On the other hand, charge disproportionation is a route to even higher but more stable oxidation states. Indeed, Fe^5+^ is obtained by charge disproportionation of less stable Fe^4+^.^18,19^ Notably, Ni^3+^ readily disproportionates to Ni^3±δ^ in rare-earth nickelates. Unusually high oxidation states are also prone to reduction, which may be accompanied by the formation of new anionic species. In the case of oxygen-rich metal oxides, phases containing molecular oxygen species may form, the most common being superoxides (O_2_^–^) and peroxides (O_2_^2–^). They are readily formed with Group 1 and 2 elements and account for numerous such phases with rich polymorphism.^34–41^ Although less common, superoxides and peroxides and their intermediates appear as oxygen-rich phases with many transition-metal elements. For example, the somewhat less stable Fe^4+^ is reduced in FeO_2_ to Fe^2+^ by the charge transfer from oxygen. This process takes place under pressure as a phase transition from oxide to peroxide, FeO_2_ → Fe­(O_2_). A similar scenario can be imagined for Cd^4+^, Hg^4+^, and Zn^4+^, where the oxidation state 4+ is too unstable and only peroxide M­(O_2_) is observed. With the aid of computational modeling, it has been demonstrated recently that the reduction via charge donation from oxygen is a means for stabilization of even more oxygen-rich transition-metal oxides. Notably, this scenario is preferred whenever the formation of a high oxidation state would require more electrons than are available in the valence shell of the metal element, as exemplified by ZrO_3_,^42^ HfO_3_,^43^ VO_4_,^44^ and Ti_2_O_5_.^45^

The above considerations drive our curiosity regarding the possible formation of novel oxygen-rich nickel oxides and their chemical identity. Is there room for the formation of novel binary nickel oxides with a higher oxygen content than that in NiO_2_? Would they host oxidation states of nickel beyond Ni^4+^? In particular, is the stabilization of Ni^5+^ with a perfectly viable electronic configuration 3d^5^ feasible in a pure oxide environment? If so, would it be inclined to disproportionation? Or is the reduction of Ni^5+^ via the formation of peroxide and superoxide phases more likely? Recognition and characterization of such phases are highly relevant to the fields of catalysis (oxidative reactions) and energy materials (photovoltaics, electrochemical water splitting, and batteries), as high- or unusual-valency transition-metal elements as well as peroxides and superoxides may form and play an important role as intermediates in important catalytic and energy-generated processes.^46,47^

In this contribution, we address the issue of possible formation of oxygen-rich binary nickel oxides by computational modeling using Density Functional Theory (DFT). As a model system, we have chosen Ni_2_O_5_. This simple stoichiometry is common to transition-metal pentoxides featuring M^5+^ species. Because of the rich polymorphism, it offers the possibility to study several structural and chemical aspects of oxygen-rich nickel oxides simultaneously, such as nickel and oxygen species, structural forms, and (partial) phases that are being preferentially formed, as well as the routes to the most likely reduction, disproportionation, or phase separation. We use two approaches for modeling Ni_2_O_5_ structures, including the substitutional approach and evolutionary algorithms for crystal structure prediction. First, the substitutional approach is used to evaluate the fitness of the crystal structures of known metal pentoxides M_2_O_5_ to accommodate Ni_2_O_5_. Within this approach, the Ni_2_O_5_ models are generated by Ni → M substitution in the crystal structures of known metal pentoxides and optimized with DFT functionals. Next, evolutionary algorithms designed for the prediction of crystal structures are used in combination with DFT calculations to predict the Ni_2_O_5_ ground-state structure. The organization of the article is as follows. We first discuss the effect of Ni → M substitution on the crystal structure and the dynamical stability of known metal pentoxides M_2_O_5_. Next, we focus on the dynamically stable Ni_2_O_5_ models obtained by the substitutional approach and the EA-predicted ground-state structure, describing their characteristic structural features (also from the perspective of known nickel oxides) and the identity of nickel and oxygen species formed in the dynamically stable models. Finally, we evaluate the thermodynamic stability of the most realistic oxygen-rich nickel oxides. These results reveal a tendency toward the reduction of Ni^5+^ via the formation of molecular oxygen species and the emergence of Ni^2+^ to Ni^4+^ oxoperoxides and/or oxosuperoxides. Notably, the formation of O_2_-decorated NiO, NiO_2_, and Ni_2_O_3_ is observed. Also, the *C*2/*c* form (the high-pressure V_2_O_5_ type structure), which is common to all known pentoxides, is found to be dynamically stable but in the case of nickel takes the form of oxosuperoxide Ni_2_O_3_(O_2_) with additional oxygen–oxygen bonds present in the otherwise intact original structure type. The predicted oxygen-rich nickel oxide phases are, however, expected to be highly thermodynamically unstable relative to the most stable nickel oxide phase, NiO.

## 2 Methods

Periodic DFT calculations were performed for Ni_2_O_5_ models in plane-wave code VASP.^48–50^ The ground-state structure was predicted with the aid of the evolutionary algorithms for crystal structure prediction implemented in the program XtalOpt.^51,52^ To successfully predict the ground-state structure, over 1400 Ni_2_O_5_ models were generated, accounting for 2, 4, 6, and 8 formula units. All EA-generated models were fully optimized with the van der Waals-corrected PBEsol functional (DFT-D3 method with Becke–Johnson damping function^53^). The models generated by Ni → M substitution in known M_2_O_5_ structure types were fully optimized using the PBEsol functional.^54^ The selected structures including the EA ground state were further optimized with van der Waals-corrected PBEsol (DFT-D3 method of Grimme^53^), Hubbard *U*-corrected PBEsol (DFT + *U* method of Dudarev^55^), and hybrid HSEsol functional.^56^ The k-mesh was set to 0.25 Å^–1^. Electronic and ionic convergence criteria were set to 10^–7^ eV and 10^–5^ eV, respectively. Lattice dynamics calculations, including phonon dispersion curves and phonon density of states, were performed on supercells using the direct method with quasiharmonic approximation implemented in the program phonopy.^57,58^ The supercells were constructed such as to meet the criteria of minimal model size (∼10 Å × 10 Å × 10 Å). Spin-polarized and electronic structure calculations were done using the Dudarev DFT + *U* approach, in which the effective Hubbard parameter, *U*_eff_ = *U* – *J*, was used to treat the electron correlations on the Ni d orbitals.^55^ The parameters *U* and *J* were set to 5 and 1 eV, respectively. The parameter *U* reported in the literature for Ni atoms in nickel oxides varies by up to 7 eV, while *U* = 5 eV was shown to be a reasonable minimum value to properly describe the magnetic and electronic structure.^59^ Chemical-bonding analysis based on periodic plane-wave (PAW) density functional theory (DFT) was performed employing computer program LOBSTER (Local Orbital Basis Suite Toward Electronic-Structure Reconstruction).^60,61^ It involved calculation of the Löwdin charges,^62^ the Crystal orbital Hamilton Population (COHP) curves,^63,64^ and the Integrated Crystal Orbital Bond Index (ICOBI).^65^ Bader charges^66–68^ were calculated using the Bader code.^68–71^ Graphics of crystal structures and Electron Localization Functions (ELFs)^72^ were created in the program VESTA.^73^

## 3 Results and Discussion

### 3.1 Crystal Structures of Transition-Metal Pentoxides

We have modeled Ni_2_O_5_ in the crystal structures of known transition-metal pentoxides. The transition-metal pentoxides show a very rich polymorphism. For the purpose of this study, we have selected all well-defined crystal structures as our starting models, leaving out their superstructures, and those that contain structural disorder, are nonstoichiometric, are stabilized by traces of other elements or have unrealistic structural features. The considered models account for three ambient-pressure V_2_O_5_ structures, including the *Pmmn* ground state (α), *Pmn*2_1_, and *Pnma* (γ′), and two high-pressure V_2_O_5_ structures quenchable at ambient conditions, *P*2_1_/*m* (β) and *C*2/*c* (δ), the latter being common to all Group 5 pentoxides (V_2_O_5_, Nb_2_O_5_, and Ta_2_O_5_) and Sb_2_O_5_. We have also accounted for three Nb_2_O_5_ structures (*A*2/*m*, *I*4/*mmm*, and *P*2_1_) and theoretically predicted the *P*1 (γ) ground-state structure of Ta_2_O_5_. All of these structures are presented in (Figure 1). They account for various metal–oxygen coordination polyhedra, including tetrahedra (Nb), square pyramids (V), and octahedra (V, Nb, Ta), which form various two-dimensional layered and three-dimensional networks via corner- and edge-sharing. The Ni_2_O_5_ models were constructed from these structures by substituting the metal elements with nickel.

**Figure 1:**
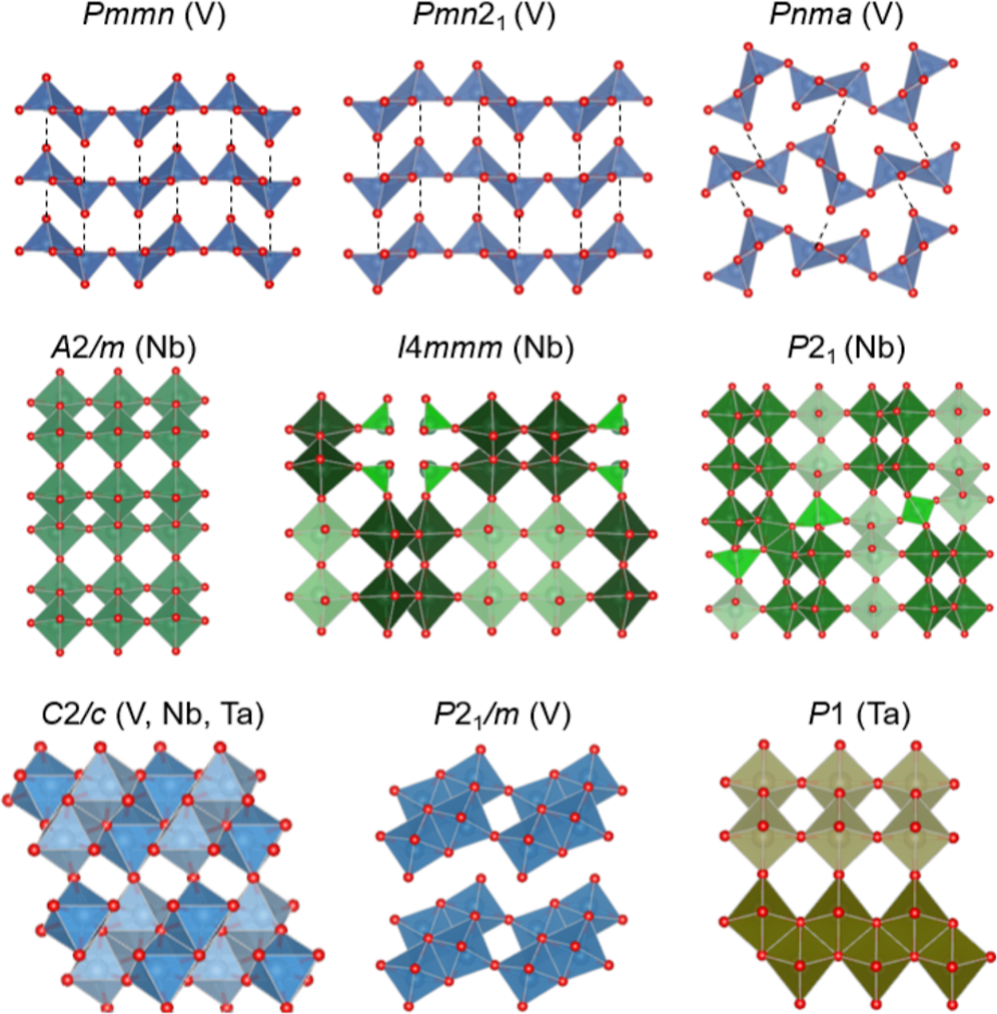
Crystal structures of transition-metal pentoxides chosen as starting models of Ni_2_O_5_. The initial Ni_2_O_5_ models were obtained by replacing the metal elements with nickel. Color code: metal–oxygen polyhedra—dark blue (V), green (Nb), light blue (V, Nb, Ta), and brown (Ta); small red balls represent O.

### 3.2 Ni-Substituted Pentoxide Structures

DFT modeling of the crystal structures of Ni-substituted metal pentoxides revealed two main factors that govern structural changes enforced by Ni substitution. First, it revealed a preference for the octahedral coordination of Ni in these structures. This is best exemplified by the Ni-substituted vanadium pentoxide models *Pmn*2_1_ and *Pnma*. The original structures are layered structures with vanadium atoms in square pyramidal [VO_5_] coordination with oxygen with V–O distances found within the range of 1.575–2.026 Å, while the longer secondary V–O contacts (2.746–2.802 Å) complete their extended distorted octahedral [VO_6_] coordination (Figure 1 top). Nickel atoms tend to increase the original 5-fold pyramidal coordination to 6-fold octahedral one in these structures by shortening the secondary metal–oxygen contacts to 1.816–1.991 Å (Figure 2, top). As a result, the layers formed by the corner- and edge-shared [VO_5_] pyramids are replaced by a three-dimensional network of corner- and edge-shared [NiO_6_] octahedra with Ni–O distances within the range of 1.737–1.991 Å. The preference for octahedral nickel is also evident from the observation of the Ni-substituted *I*4/*mmm* Nb_2_O_5_ structure, where the original [NbO_4_] tetrahedra with Nb–O distances equal to 2 × 1.63 and 2 × 1.97 Å (Figure 1 middle) are replaced by edge-shared [NiO_6_] octahedra with distances within the range of 1.823–2.015 Å by shortening the secondary metal–oxygen contacts from 2.45 to 2.015 Å (Figure 2, middle).

**Figure 2:**
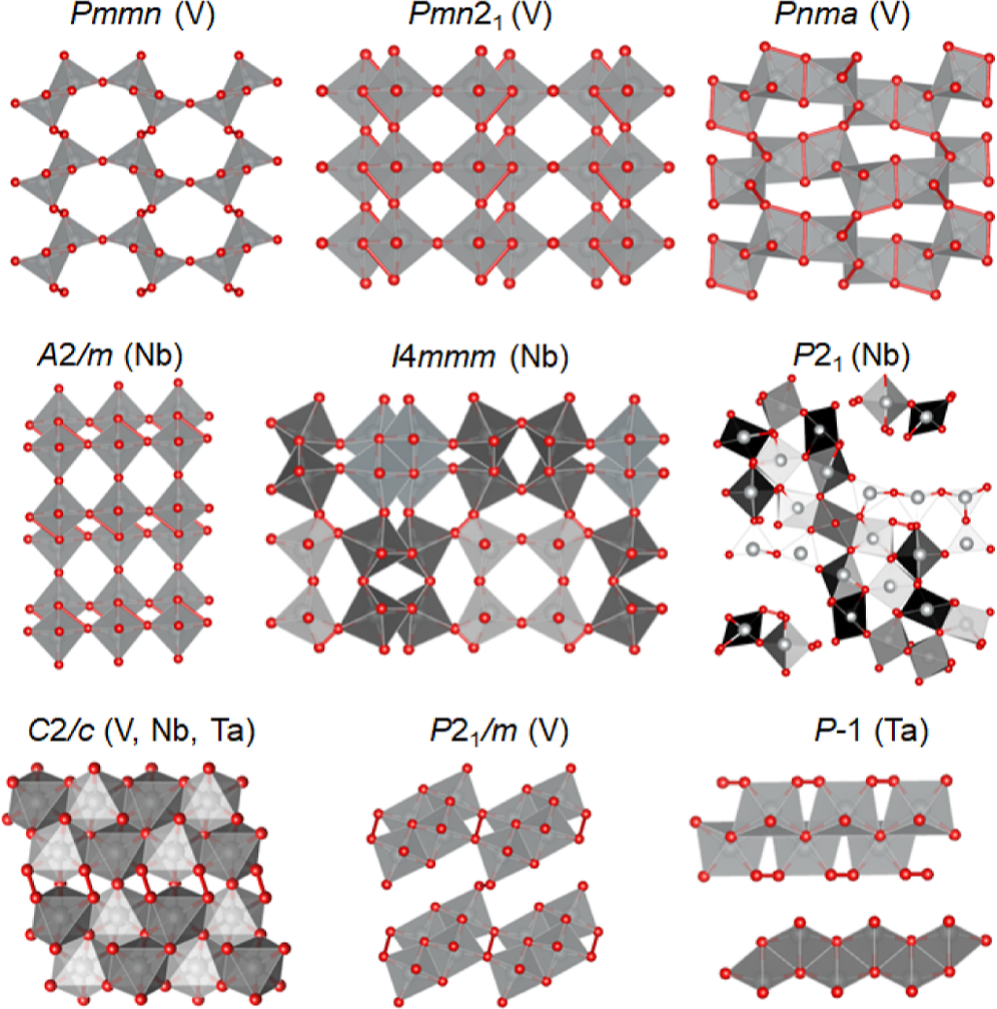
Ni_2_O_5_ DFT-optimized in structure types of known transition-metal pentoxides presented in Figure 1. Unusually short Oxygen–oxygen contacts (≤2.3 Å) are highlighted by red connectors. Color code: nickel–oxygen polyhedra—shades of gray, small red balls—O.

Second, there is a strong tendency toward the formation of molecular oxygen species, which furthermore dominates the preference for octahedral nickel. This tendency manifests itself by the pronounced shortening of the oxygen–oxygen contacts highlighted in Figure 2 by the red connectors. They account for two characteristic regions of interatomic O–O separations, including very short ones (1.29 to 1.37 Å) and highly atypical distances (1.86 to 2.35 Å). The former values are typical for molecular oxygen species in which the intramolecular O–O distances usually range from ∼1.2 Å (O_2_) to ∼1.4 Å (O_2_^–2^). The latter are much longer than the bonding distances within molecular oxygen species but considerably shorter than the corresponding oxygen–oxygen distances in the original structures or transition-metal oxides in general (≥2.4 Å). Typical oxygen–oxygen distances in metal oxides are usually comparable or longer than twice the crystal ionic radius of O^2–^. Smaller values are extremely rare and are observed only in simple oxides with small p elements such as NaNO_3_ (2.15 Å) and CaCO_3_ (2.22 Å) or the extremely dense form of SiO_2_ stishovite (2.16 Å).^74^ Regarding Ni_2_O_5_, formation of the molecular O_2_ species is observed in three models, including *C*2/*c*, *I*4/*mmm*, and *P*2_1_, while the atypical O–O distances are present in almost all models.

Figure 3 reveals that the Ni_2_O_5_ models with atypical oxygen–oxygen distances have considerably higher energies than those in which all short oxygen–oxygen distances are in line with the formation of molecular oxygen species. There is an approximately 70 kJ/mol of Ni_2_O_5_ energy difference between the two groups of models. This observation, together with the considerations mentioned above, implies that the Ni_2_O_5_ models with atypical oxygen–oxygen distances do not reflect optimal structures. In fact, atypical oxygen–oxygen distances were found to be the consequence of symmetry constraints defined by the structure types. Once these constraints were released, molecular oxygen species were formed, and the total energy of the models was severely reduced by ≥70 kJ per mol of Ni_2_O_5_ as confirmed by DFT calculations (Figure 3) as well as by DFT + *U* calculations (see Figure S6 in Supporting Information), in which we accounted for electron correlations and the magnetic character of nickel atoms. Both methods, DFT and DFT + *U*, provided qualitatively comparable results.

**Figure 3:**
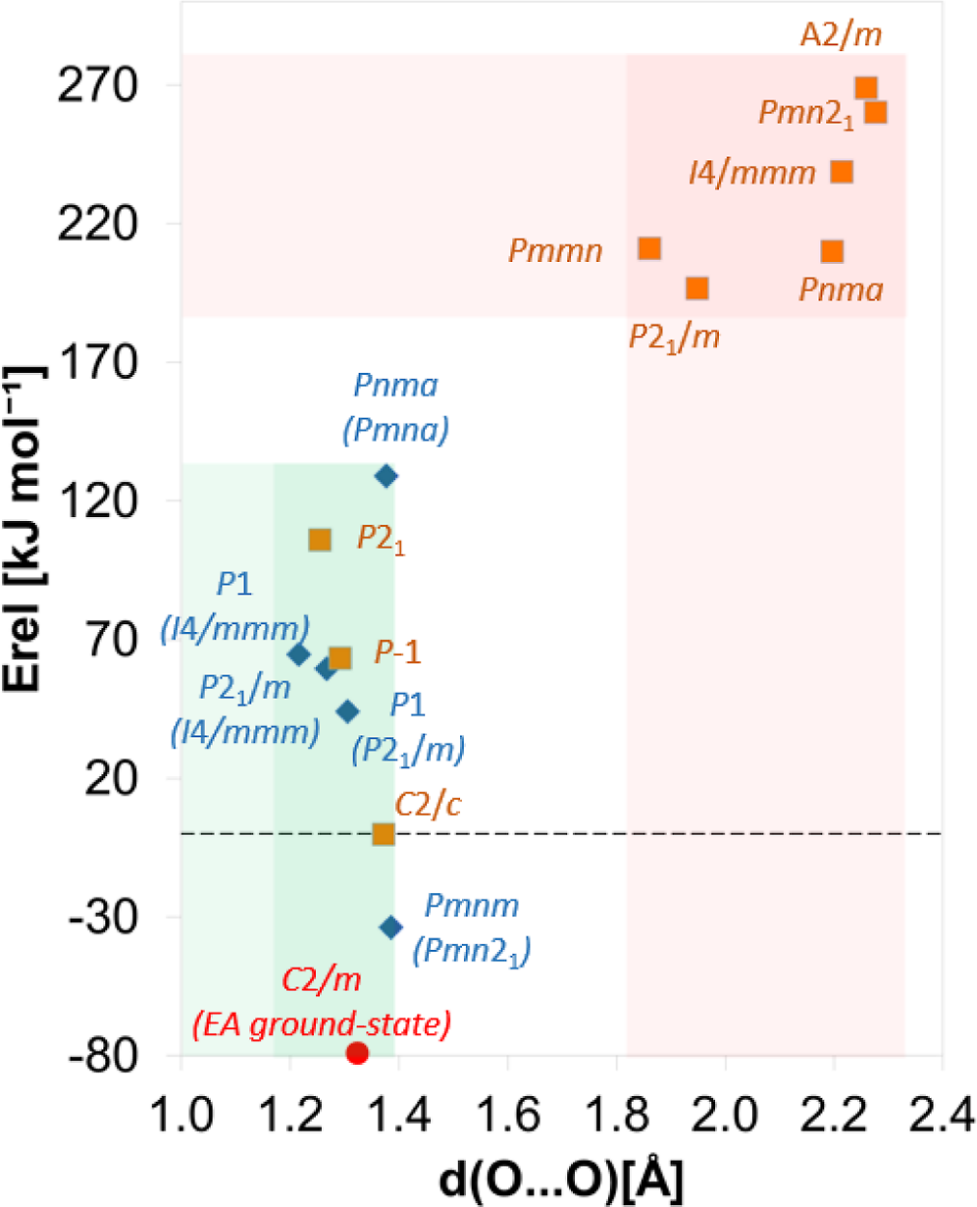
Total DFT energies of optimized Ni_2_O_5_ models calculated relative to the *C*2/*c* solution, which has the lowest energy after M → Ni substitution, and plotted as a function of the type of oxygen–oxygen distance present. Two sets of data are shown for the M → Ni-substituted structures: results from optimizations during which the original symmetries were kept (orange squares) and during which the symmetries were broken (blue diamonds). In the case of the symmetry-broken models, the original symmetries prior to the symmetry-breaking are provided in parentheses. The red stripes highlight the models with atypical oxygen–oxygen contacts. The green stripes highlight the models in which all short oxygen–oxygen contacts are consistent with the formation of molecular oxygen species. The EA-predicted Ni_2_O_5_ ground-state structure is also shown (red circle). Similar results were obtained with spin-polarized DFT + *U* (see Figure S6 in Supporting Information).

Note that in the case of the structures *Pmn*2_1_, *Pmna*, *A*2/*m*, *I*4/*mmm*, and *P*2_1_/*m*, the atypical oxygen–oxygen distances concern a considerable part of the oxygen sublattice (Figure 2). These models have experienced the most severe structural changes after the release of the symmetry constraints. The drive for the formation of molecular oxygen species has led to complete destruction of the crystal structure of most of them, making them completely unsuitable for modeling Ni_2_O_5_. In particular, the formation of molecular oxygen species in the *I*4/*mmm* structure was accompanied by the formation of numerous metal–oxygen coordination polyhedra (3- to 6-fold) without any apparent structural leitmotif (see Figure S7 in Supporting Information). A similar collapse of the crystal structure was observed for the *P*2_1_ structure (compare Figures 1 and 2), where the original symmetry did not pose any constraints on the atomic positions of oxygen. In the case of the *P*2_1_/*m* structure, the formation of molecular oxygen species was accompanied by the dissociation of the metal–oxygen layers into complex chains consisting of edge-shared [NiO_6_] octahedra and [NiO_5_] square pyramids and linked by the molecular O_2_ units into a three-dimensional *P*1 crystal lattice (Figure S9 in Supporting Information), which was found to be dynamically unstable (Figure S10 in Supporting Information). On the other hand, atypical oxygen–oxygen contacts concern only a small part of the oxygen sublattice in structures *Pmmn* and *C*2/*c*. In the *Pmmn* structure, they are limited to interlayer interactions that involve terminal oxygen atoms. Removal of the symmetry constraint leads to a *P*2_1_/*m* model, in which molecular oxygen–oxygen bridges are formed between the neighboring layers via dimerization of the terminal oxygen atoms. However, oxygen dimerization enforces severe puckering of the layers, which were also found to be dynamically unstable (Figure S2 in Supporting Information). The *C*2/*c* structure is the only model that does not pose any symmetry constraints on the formation of molecular oxygen species, while their formation requires only slight adjustments of the atomic positions (compare Figures 1 and 2). Furthermore, the resulting structure has the lowest energy among the least modified structures (including *P*2_1_/*m* and *P*1̅, Figure 3) and is dynamically stable (see Figure S1a in Supporting Information). This result suggests that Ni_2_O_5_ could in principle be obtained in the *C*2/*c* structure, which is virtually identical to the *C*2/*c* V_2_O_5_ type, additionally enriched by the interlayer molecular oxygen contacts. This result is further supported by the fact that the *C*2/*c* structure is the most common structure type among metal pentoxides (V, Nb, Ta, and Sb).

Importantly, in *Pmn*2_1_ (and *A*2/*m*), relaxation of the atypical oxygen distances resulted in a reconstruction to a novel dynamically stable layered structure with *Pmnm* symmetry (Figure 4) energetically preferred even over the *C*2/*c* structure (Figure 3). This shows that the *C*2/*c* structure should be expected as the metastable polymorph of Ni_2_O_5_ similarly as in the case of the well-known transition-metal pentoxides (V, Nb, Ta, and Sb), but at the same time, novel lower-energy Ni_2_O_5_ structures should be expected. Regarding the latter, there seems to be a preference for novel layered Ni_2_O_5_ structures with molecular O–O bridges, as further exemplified by the Ni-substituted *P*1 model, which emerges as a dynamically stable *P*1̅ structure (Figure 2, bottom and Figure S1b in Supporting Information) after complete reconstruction of the original structure (Figure 1, bottom). The crystal structures of the new layered polymorphs will be discussed in detail below.

**Figure 4:**
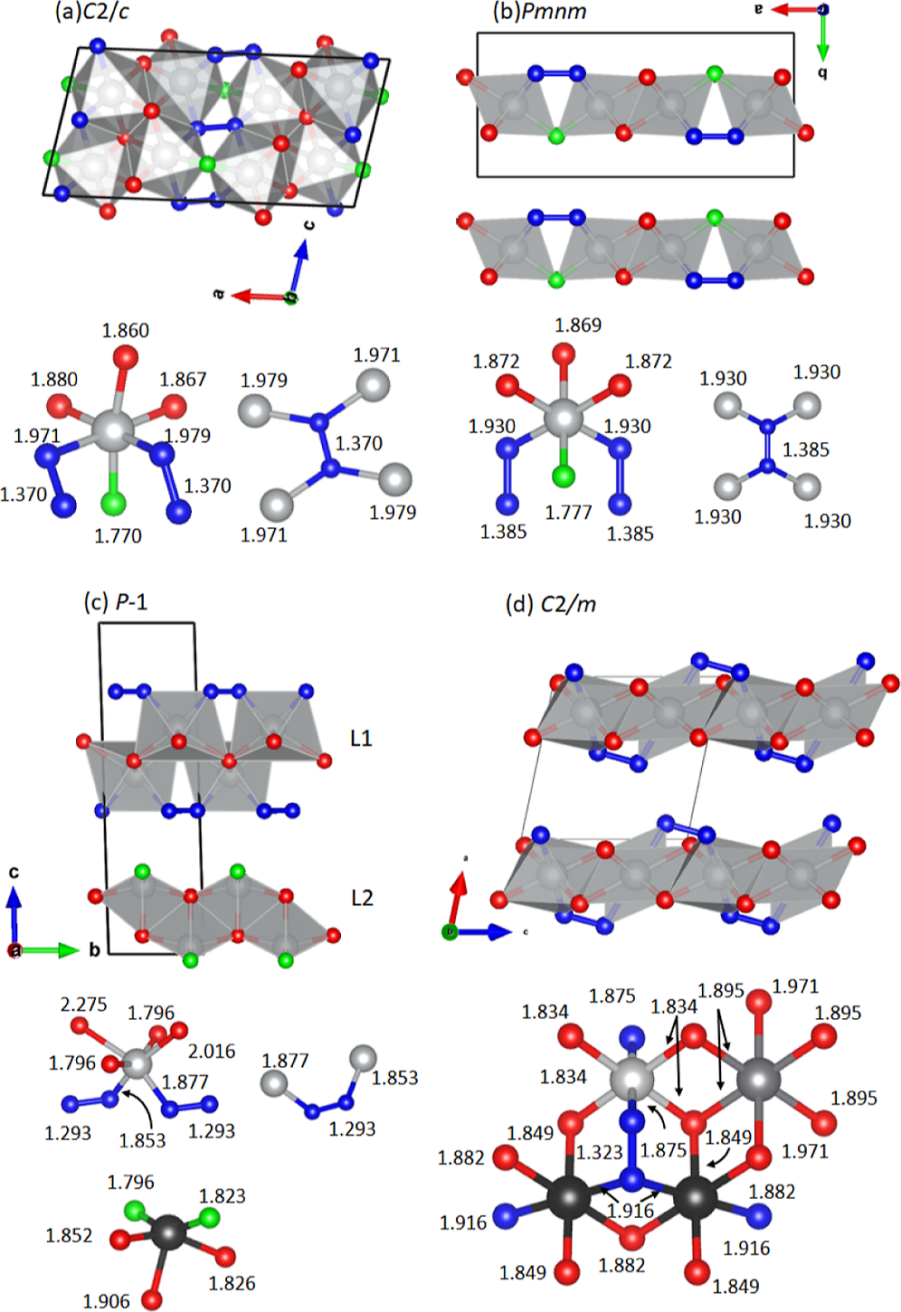
DFT Crystal structures of dynamically stable Ni_2_O_5_ models, highlighting polyhedral connectivity and the coordination sphere of Ni and O_2_ species: (a) *C*2/*c*, (b) *Pmnm*, (c) *P*1̅ (L1 = NiO_3_, L2 = NiO_2_), and (d) *C*2/*m* (EA ground state). Legend: Ni: shades of gray; O_2_ species: blue; 2-fold O: green; 3- and 4-fold O species: red. In (d) three independent Ni sites are distinguished.

### 3.3 Ground-State Structure from EA

The above conclusions reached using the substitutional approach are in agreement with our subsequent calculations, which employed evolutionary algorithms for crystal structure prediction. All structures generated with EA contained molecular oxygen species confirming the preference for their formation in Ni_2_O_5_. Furthermore, the ground-state structure predicted with the aid of EA is an oxygen-enriched CdI_2_-type *C*2/*m* structure (Figure 4d) similar to the *Pmnm* structure (Figure 4b) obtained from the less computationally demanding substitutional approach as the lowest-energy structure. The EA-predicted *C*2/*m* ground-state structure was predicted to be dynamically stable (Figure S1c in Supporting Information). It differs from *Pmnm* only by different incorporation of O_2_ species within the CdI_2_ layers. This change results in a further reduction in the total energy by 50 kJ per mole of Ni_2_O_5_ relative to the energy of the *Pmnm* structure (Figure 3, red circle). The crystal structure of the *C*2/*m* ground state is discussed in more detail below, together with the dynamically stable structures obtained by the substitutional approach.

### 3.4 Dynamically Stable Ni_2_O_5_ Structures

The above results have revealed four dynamically stable candidates for the crystal structures of Ni_2_O_5_ (Figure 4). They account for the *C*2/*c* model, which is intimately related to the known pentoxide structures of the same *C*2/*c* symmetry, and three novel layered structures, *Pmnm* and *P*1̅ and the EA-predicted *C*2/*m* ground-state structure. All four structures were optimized on several levels of DFT theory, accounting for van der Waals corrections, Hubbard *U* correction, and hybrid DFT. They confirm that the EA *P*1̅ structure is the ground state and in the following order in terms of increasing total energy: *C*2/*m*, *Pnmn*, *C*2/*c*, and *P*1̅ (Table S2 in Supporting Information). The impact of magnetic configurations on relative stability was also partly considered. The structural features of the four models, such as significant octahedral edge-sharing and low Ni–O–Ni angles (usually below 110°), indicate that ferromagnetic (FM) ordering will be favored over antiferromagnetic ones. Tests performed for one of the dynamically stable models, *C*2/*c*, have confirmed these assumptions (Figure S11 and Table S7 in Supporting Information).

In the *C*2/*c* structure, all characteristic features of the original *C*2/*c* prototype are preserved. The main building block of the *C*2/*c* structure (Figure 4a) is a dimeric unit formed of two nickel–oxygen [NiO_6_] octahedra that share a common edge. The dimers are organized into one-dimer-thick layers (in *bc* plain) and connected by corners within and between the layers. The structure can also be understood as a distorted *hcp* oxygen lattice with metal cations occupying the distorted octahedral voids, and the dimer-containing layers represent rutile-type two-dimensional fragments. In the Ni-substituted structure, the [NiO_6_] octahedra are more regular than those in the original structure. This is evident from the narrower range of the Ni–O distances. In the Ni-substituted structure, the Ni–O distances are found within the range 1.77–1.98 Å, while the corresponding M–O distances in the original *C*2/*c* pentoxides range between 1.64–2.15 Å (V–O), 1.78–2.20 Å (Nb–O), and 1.67–2.24 Å (Ta–O). The only novel feature of the Ni-substituted *C*2/*c* structure is the formation of molecular O_2_ units. The oxygen–oxygen bonds develop between the least-coordinated oxygen atoms (as typically observed in metal oxides). In the *C*2/*c* structure, the O_2_ dimers with an O–O bonding distance of 1.37 Å form between 2-fold oxygen atoms from neighboring rutile-like layers, where they gain end-on transcoordination with four Ni atoms, two from both O_2_ ends. Similar geometry was predicted for the high-pressure form of VO_4_.^44^ Here, the emerging layer-bridging O_2_ units are found in a transcoordination by two Ni atoms.

The new layered *Pmnm* structure (Figure 4b) consists of infinite two-octahedra-thick CdI_2_-type stripes of edge-shared octahedra. The neighboring stripes are oriented to each other face-on within layers and are connected by corner-sharing and by molecular O_2_ bridges (1.385 Å). Each O_2_ unit coordinates four Ni atoms, as is also the case in the *C*2/*c* structure, but the nickel atoms are in ciscoordination, while they are in transcoordination in the *C*2/*c* structure. The layers interact by weak dispersive interactions, as indicated by the shortest interlayer O···O_2_ contacts of 3.16 Å that involve 2-fold monooxygen anions and molecular O_2_ species. The Ni–O distances are found in the *Pmnm* structure within the range of 1.78–1.93 Å comparable to the *C*2/*c* structure.

The EA-predicted *C*2/*m* ground-state structure (Figure 4d) is closely related to the *Pmnm* structure. They both consist of CdI_2_-type layers of edge-shared octahedra with excess oxygen atoms. In each case, oxygen atoms enter the layer at a different site, leading to different distortions of the layers and the geometry of the molecular O_2_ atoms within them. In the *Pmnm* structure, there is one independent Ni site and all [NiO_6_] octahedra share four edges with their neighbors. In the *C*2/*m* structure, there are three independent Ni sites and their corresponding [NiO_6_] octahedra share six, four, and two edges with their neighbors, respectively. The O–O bonding distance in the *C*2/*m* structure is slightly shorter (1.32 Å) when compared to *Pmnm* (1.39 Å). The O_2_ species gain end-on ciscoordination with three Ni atoms, two at one end, and the remaining one at the other end, while only two independent Ni sites are coordinated by O_2_ units (3/4 of all Ni atoms). The third independent Ni site retains a coordination environment reminiscent of the undistorted Cd_2_-type layers. In effect, while the *Pmnm* layers can alternatively be viewed as polymerized CdI_2_-type stripes additionally bridged by O_2_ units, *C*2/*m* layers can alternatively be viewed as polymerized rutile-like chains additionally bridged by O_2_ units (the layers of both structures are compared in Figure S8 in Supporting Information). The Ni–O distances in the *C*2/*m* structure (1.85–1.97 Å) are slightly longer than in the *Pmnm* structure. The layers in the *C*2/*m* structure are puckered, while they remain flat in the *Pmnm* structure similarly to the original CdI_2_-type structure.

Lastly, the new layered *P*1̅ structure consists of two distinct layers, nominally [NiO_2_] and [NiO_3_] (Figure 4c). The structure of the [NiO_3_] layer can be described as consisting of a puckered rock salt [NiO] sheet decorated from both sides by O_2_ dimers (1.29 Å). The O_2_ dimers complete the distorted octahedral coordination of the Ni atoms. Each unit of the O_2_ coordinates two Ni atoms in transcoordination. The [NiO_2_] layer consists of infinite chains of edge-shared [NiO_5_] units (propagating a long *b*). The chains are polymerized into layers by sharing the remaining corners along the *a* direction. The [NiO_5_] units can be described as distorted trigonal bipyramids with Ni atoms shifted from the central position toward one of the edges of the base. The [NiO_2_] and the [NiO_3_] layers interact by weak dispersive O···O_2_ interactions, as suggested by the shortest interlayer contacts of 2.87 Å, which form between the 2-fold oxygen and O_2_ species of the two layers, respectively. The new *P*1̅ structure can thus be described as a van der Waals heterostructure of two phases, nominally NiO_2_ and NiO_3_. The Ni–O distances are found in the *P*1̅ structure within the range of 1.796–2.275 Å in the [NiO_3_] layer and 1.796–1.906 Å in the [NiO_2_] layers.

It is noteworthy that all three layered structures, *Pmnm*, *C*2/*m*, and *P*1̅, could be considered oxygen-rich variations of the already well-known nickel oxide phases, namely NiO and NiO_2_. The *Pmnm* and *C*2/*m* structures represent the known NiO_2_ layered form with an extra O atom per two NiO_2_ formula units within the layers. On the other hand, O_2_-decorated rock salt NiO monolayers and layers of a potentially novel NiO_2_ form are present in the *P*1̅ structure.

In all four Ni_2_O_5_ structures, molecular O_2_ species are present, as indicated by the interatomic distances. Their formation was confirmed by analysis of the calculated electron localization functions (ELFs). In Figure 5, ELF maps are presented for the four structures. In each case, a local maximum is observed in the scalar distribution of the ELF (localized electron density) along the O–O connector. These local maxima are highlighted in Figure 5 by an arrow and defined as bonding attractors in the original work.^72^ Their presence confirms the formation of a covalent O–O bond and thus the formation of a molecular O_2_ species.

**Figure 5:**
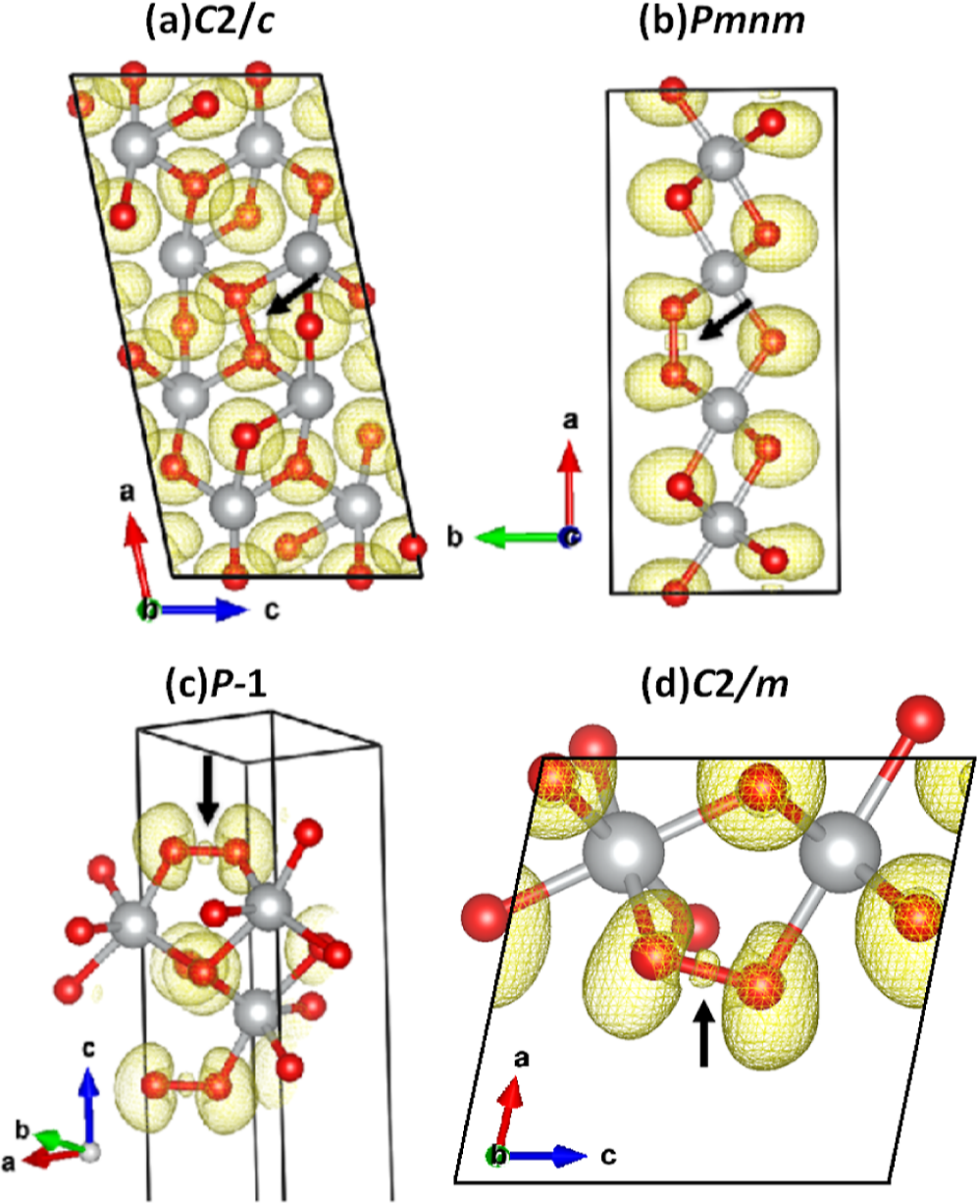
Electron localization functions (ELFs) calculated with DFT for selected Ni_2_O_5_ models: (a) *C*2/*c*, (b) *Pmnm*, (c) *P̅*1, and (d) *C*2/*m*. The presence of a local maximum in the ELF scalar distribution along the O–O connector (localized electron density) is highlighted by an arrow. The isosurface value was set to 0.68.

### 3.5 Superoxide or Peroxide?

To get deeper insights into the character of molecular O_2_ species, which are being formed in the predicted Ni_2_O_5_ models presented above, we next confronted the structural analysis with the analysis of Bader^66–68^ and Löwdin charges,^62^ O–O stretching frequencies, Crystal Orbital Hamilton population (COHP)^63,64^ analysis, and Integrated Crystal Orbital Bond Index (ICOBI).^65^

As noted above, the O–O bonding distances in the Ni_2_O_5_ models are calculated within the range of 1.29–1.37 Å. In the case of the *C*2/*m* and *P1̅* structures, they are found to be close to 1.3 Å while those in *C*2/*c* and *Pmnm* structures are close to the value of 1.4 Å. The former value is typical for an O_2_^–^ anion, while the latter is typical for an O_2_^2–^ anion. These results suggest the formation of superoxide species in the former structures and peroxide species in the latter two structures (Table 1). The calculated O–O stretching vibrations are consistent with the above result. Their values were calculated within the range of 850–904 cm^–1^ for the *C*2/*c* and *Pmnm* models and 1077–1235 cm^–1^ for the *C*2/*m* and *P1̅* models. The lower frequencies are characteristic of the peroxide species, while the higher values are characteristic of the superoxide species (Table 1).

**Table 1:** List of Interatomic Distances within the O–O Bridges and Stretching O–O Frequencies Calculated with DFT-PBEsol for the Dynamically Stable Ni_2_O_5_ Models^a^

compound	SPGR	*d*_(O–O)_ [Å]	*v*_O–O_ [cm^–1^]
Ni_2_O_5_	*C*2/*c*	1.371	850^*a*^ (Bg)
			863^*s*^ (Ag)
	*Pmnm*	1.385	889^*a*^ (B2u)
			904^*s*^ (Ag)
	*C*2/*m*^*EA*^	1.324	1100 (Au)
			1077 (Ag)
	*P1̅*	1.291	1227^*a*^
			1235^*s*^
O_2_		1.2	1300
O_2_^–^		1.3	1100
O_2_^2–^		1.44	800
peroxides		1.26–1.62	738–876
superoxides		1.192–1.383	1100–1200

a The respective values taken from the literature for the molecular oxygen species as well as superoxides and peroxides of Groups I and II elements are provided for comparison.^34–41^

Regarding Bader charges, it is expected that the formal charge per oxygen site in the superoxide and peroxide species is reduced by a factor of 2 and 4, respectively, relative to the charge on the O^2–^ anion. This is indeed the case for our Ni_2_O_5_ models, as witnessed by the calculated Bader charges. In Table 2, the Bader charges calculated for Ni and O atoms of the selected Ni_2_O_5_ models are listed together with the corresponding chemical formula, which highlight the presence of the O_2_ species. The Bader charge analysis revealed that in the *C*2/*c* and *Pmnm* models, the average charge on monooxygen species is −0.75*e* ^–^, while the charge on oxygen atoms involved in the O–O bridges is close to −0.38*e* ^–^. In the case of the *C*2/*m* and *P*1̅ structures, the charge on the oxygen atoms involved in the O–O bridges is even lower and amounts to −0.29*e* ^–^ and −0.24*e* ^–^, respectively, while the average charge on the monooxygen species gains values between −0.80*e* ^–^ and −0.95*e* ^–^. For comparison, the Bader charges calculated for known nickel oxides, namely, NiO and NiO_2_, are also shown in Table 2 (bottom). The Bader charges on monooxygen anions in the Ni_2_O_5_ models are comparable to the corresponding value obtained for NiO_2_ (−0.77*e* ^–^). The only exceptions are oxygen atoms, which are part of the van der Waals [NiO_3_] sublayers in the *P*1̅ model. Their Bader charges are considerably larger (−0.95*e* ^–^) and are more comparable to those in the NiO structure (−1.17*e* ^–^). The latter result is consistent with the structural analysis of the [NiO_3_] layers, which revealed that they consist of [NiO] sheets decorated from both sites by the O_2_ species. Importantly, Bader analysis suggests the formation of peroxygen (O_2_)^2–^ species in *C*2/*c* and *Pmnm* and superoxygen (O_2_)^−^ anions in the *C*2/*m* and *P1̅* models. The same conclusion is reached from the analysis of the Löwdin charges, which provide slightly lower values (Table S1 in the Supporting Information).

**Table 2:** Bader Analysis (DFT-PBEsol) for Selected Ni_2_O_5_ Models, Including the Original V_2_O_5_ *C*2/*c* Compound and Ni_2_O_5_ in the Original (Unrelaxed) *C*2/*c* Type (V^*Ni*^_2_O_5_): O^bridge^—O_2_ Species, O—Monooxygen, Ni—Nickel Species

oxide	SPGR	formula	O^bridge^	O	Ni
V_2_O_5_	*C*2/*c*	V_2_O_5_	–0.76	–0.92	2.16
V^*Ni*^_2_O_5_	*C*2/*c*	Ni_2_O_5_	–0.56	–0.68	1.58
Ni_2_O_5_	*C*2/*c*	Ni_2_O_3_(O_2_)	–0.37	–0.75	1.52
	*Pmnm*	Ni_2_O_3_(O_2_)	–0.39	–0.74	1.52
	*C*2/*m*^*EA*^	Ni_2_O_3_(O_2_)	–0.29	–0.80	1.49–1.51
	*P*1̅	[NiO(O)_2_]	–0.24	–0.95	1.42
		[NiO_2_]	NA	–0.74	1.49
NiO	*Fm*3̅*m*		NA	–1.17	1.17
NiO_2_			NA	–0.77	1.54

To separate the effect of metal substitution in the original pentoxide structures from subsequent structural changes caused by the formation of the O_2_ species, we have additionally calculated Bader charges for the original metal pentoxide structures before and after Ni substitution (Table 2, top). The most suitable candidate for this analysis is the V_2_O_5_ *C*2/*c* structure because the relevant structural changes enforced by Ni substitution are limited to the formation of the O_2_ species. Replacement of vanadium with nickel resulted in a pronounced reduction in the charges on all atoms. Such an overall charge reduction is consistent with increased covalent character, which is to be expected when moving toward heavier 3d elements. Subsequent relaxation of the Ni-substituted structure resulted in a large redistribution of the charge among the molecular and single oxygen anions and a decrease in the charge on the Ni atoms. The charge on the oxygen anions involved in the formation of molecular O_2_ species has been further reduced, and on the remaining singular oxygen anions it has been increased. In particular, the charge on molecular oxygen has decreased by a factor of 2 relative to the charge on the corresponding oxygen atoms in the original V_2_O_5_ compound further confirming formation of a peroxide anion in the Ni-substituted and relaxed *C*2/*c* structure.

The O_2_ bonding lengths, stretching frequencies, and Bader charges suggest the formation of peroxide anions (O_2_^–2^) in *C*2/*c* and *Pmnm* models and superoxide anions (O_2_^–^) in the *C*2/*m* and *P1̅* models. The formation of a peroxide anion implies completely occupied molecular O_2_ π* antibonding orbitals, while in the case of a peroxide anion only 3/4 of the molecular O_2_ π* orbitals should be populated. Spin-resolved COHP analysis performed for the O_2_ species reveals a partial depopulation of the O_2_ π* orbitals in all four cases, suggesting that the molecular O_2_ species are in a state intermediate between superoxide and peroxide (Figure 6). This conclusion is partly supported also by the calculated ICOBI values, which indicate Lewis bond order. The bond order of the isolated O_2_^–^ and O_2_^2–^ anions is equal to 1.5 and 1, respectively. The ICOBI values calculated for *C*2/*c* (1.0) and *Pmnm* (1.0) suggest the formation of peroxide anions in the two structures. The ICOBI values calculated for *C*2/*m* (1.1) and *P*1̅ (1.2), on the other hand, indicate that the corresponding O_2_ anions are in a state intermediate between superoxide and peroxide.

**Figure 6:**
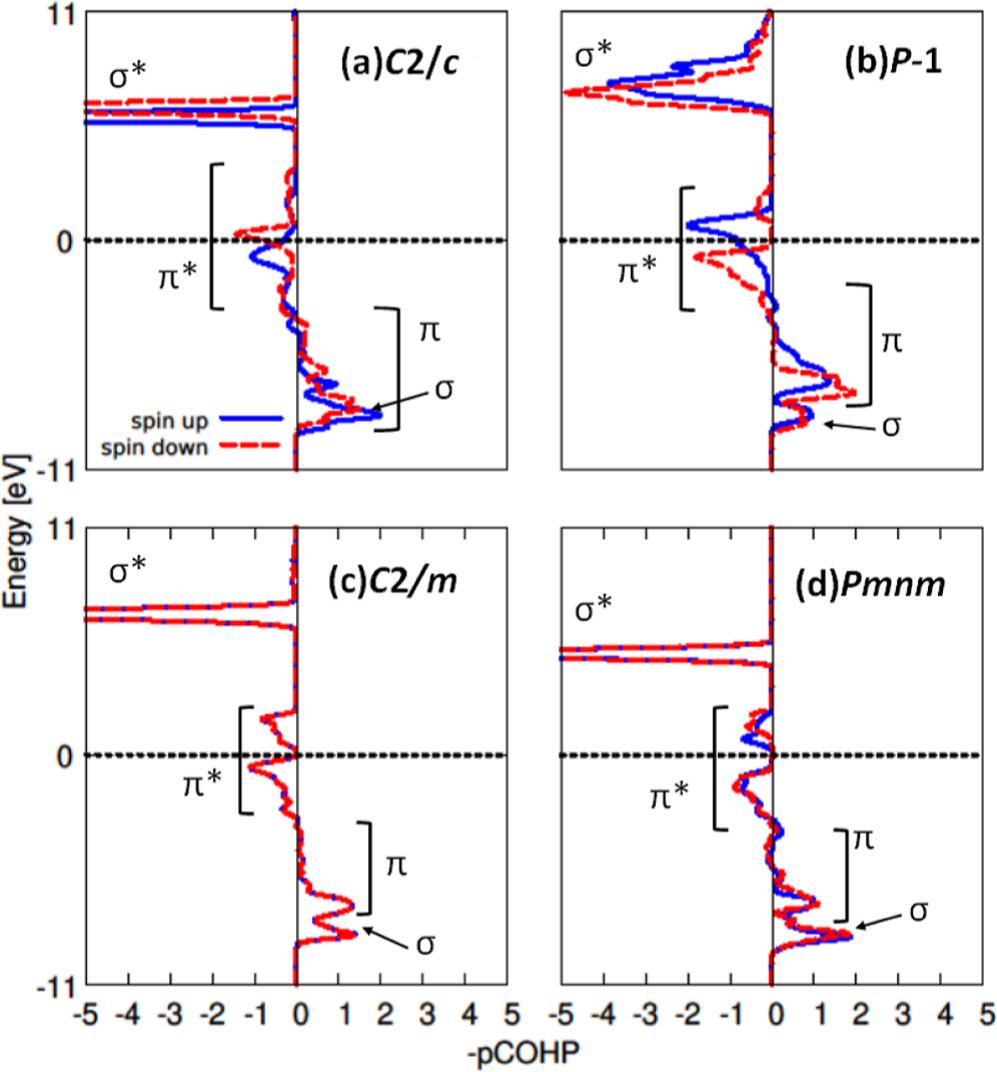
COHP analysis of bonding situation within O_2_ dimers calculated with DFT + *U* for four selected Ni_2_O_5_ models: *C*2/*c* (a), *P*1 (b), *C*2/*m* (c), and *Pmnm* (d). In each panel, the two curves represent spin-up and spin-down contributions.

Another property that distinguishes the superoxide anion from the peroxide anion is the magnetic moment. For a peroxide anion with fully occupied O_2_ π* orbitals, zero magnetic moment is expected, while for a superoxide anion with one hole in the doubly degenerate π* orbitals 1 m_*B*_ from the one unpaired spin is expected. The large magnetic moments per O_2_ species in the *P*1̅ (0.8 m_*B*_) and vanishing ones in the *Pmnm* structure (0.06 m_*B*_) suggest the presence of superoxide- and peroxide-like species, respectively, which correlate well with the above results. On the other hand, the magnetic moments per O_2_ species in the *C*2/*c* (0.48 m_*B*_) and *C*2/*m* (0.00 m_*B*_) structures indicate more peroxide-like anions in the former and superoxide-like ones in the latter, contrary to the above analysis. The presence of a nonzero magnetic moment as well as depopulated O_2_ π* in peroxide anions in Ni_2_O_5_ models may be explained by higher hybridization of the metal and O_2_ states, (*i.e.* increased covalency of M–O_2_ bonds) than in alkali and alkali-earth peroxides with purely ionic M–O_2_ bonds. Detection of superoxide and peroxide species on the basis of magnetization thus becomes less meaningful in the transition-metal oxides. The increased Ni­(d)/O_2_(*p*) hybridization and increased covalency were confirmed by atom-projected electronic density of states and ELF analysis, respectively (Figures S4 and S5 in Supporting Information).

### 3.6 Identification of Nickel Species

The formation of molecular O_2_ species in Ni_2_O_5_ implies a reduction in the formal oxidation state of nickel below Ni^5+^. The extent of this reduction is reflected by the number and type of molecular oxygen species that are being formed. The above identification of the molecular oxygen species present in the Ni_2_O_5_ models suggests the following average oxidation states on the Ni atoms, which are also summarized in Table 3. The structures *C*2/*c*, *Pmnm*, and *C*2/*m* contain one O_2_ species per formula unit, Ni_2_O_3_(O_2_). Considering the formation of a peroxide anion in the former two structures and a superoxide anion in the latter one suggests the presence of Ni^4+^ in *C*2/*c*, *Pmnm*, and Ni^3.5+^ in *C*2/*m*. Considering the formation of a superoxide anion in the NiO­(O_2_) layers of the *P*1̅ heterostructure, which consists of chemically distinct layers, NiO­(O_2_) and NiO_2_, the presence of Ni^3+^ and Ni^4+^ is expected in the two layers, respectively. The fractional average oxidation state of Ni^3.5+^ in *C*2/*m* implies the presence of multiple Ni atoms with different oxidation states. This is consistent with the presence of three independent Ni sites in the *C*/2*m* structure. Further support comes from the observation of the Bader charges. Slightly different values equal to 1.49*e* ^–^, 1.50*e* ^–^, and 1.51*e* ^–^ are obtained for the three distinct Ni atoms. On the other hand, these values are much closer to 1.52*e* ^–^ obtained for Ni^4+^ in structures *C*2/*c* and *Pmnm* than to 1.42*e* ^–^ calculated for Ni^3+^ of NiO­(O_2_) layers of the *P*1̅ heterostructure (Table 2). Recalling also the calculated bond order of 1.1, which is more reminiscent of a peroxide than a superoxide species, these observations offer an alternative interpretation for the *C*2/*m* structure—oxoperoxide of Ni^4+^. It should also be noted that the Bader charge on Ni atoms in all models is slightly lower than in the known nickel dioxide Ni^4+^O_2_ compound (1.54*e* ^–^) and simultaneously considerably higher than in the known nickel monoxide Ni^2+^O compound (1.17*e* ^–^). This comparison further suggests the formation of Ni species ranging from Ni^3+^ to Ni^4+^. However, these comparisons should be considered with some reservations, since the Bader charges also reflect the distinct character of bonding and the topology of bonds.

**Table 3:** The Identity of Molecular O_2_ Species in the Selected Ni_2_O_5_ Models Deduced from the Analysis of DFT-Calculated O_2_ Bonding Distances, Stretching Frequencies, Bader Charges, and Average Oxidation State of Nickel Expected in Each Case, Together with DFT + *U* Calculated Absolute Values of Magnetic Moment per Ni and O_2_ Species

SPGR	formula	O_2_	Ni	Ni [μ_B_]	O_2_ [μ_B_]
*C*2/*c*	Ni_2_O_3_(O_2_)	(O_2_)^2–^	Ni^4+^	0.65	0.46
*Pmnm*	Ni_2_O_3_(O_2_)	(O_2_)^2–^	Ni^4+^	0.25	0.06
*C*2/*m*^*EA*^	Ni_2_O_3_(O_2_)	(O_2_)^1–^	Ni^3.5+^	0	0
*P*1̅ (L1)	[NiO(O)_2_]	(O_2_)^1–^	Ni^3+^	0.85	0.72
*P*1̅ (L2)	[NiO_2_]	NA	Ni^4+^	0.92	NA

### 3.7 Thermodynamical Stability

Thermodynamic stability of the Ni_2_O_5_ models was evaluated on the basis of the calculated energy of formation ([Disp-formula eq1]) and energy of the reaction (2)

1 $$2Ni+5{\left(O_{2}\right)}_{1/2}\to Ni_{2}O_{5}$$

2 $$2NiO+3/2O_{2}\to Ni_{2}O_{5}$$

The energy of formation ([Disp-formula eq1]) indicates the stability of Ni_2_O_5_ relative to the constituent elements in their standard state. For this purpose, fcc Ni and the low-temperature antiferromagnetic monoclinic structure of molecular O_2_ were chosen.

The energy of the reaction (2) assesses the stability of Ni_2_O_5_ relative to the decomposition to the most stable nickel oxide, the antiferromagnetic NiO, and the antiferromagnetic monoclinic molecular oxygen. The energies of both reactions calculated for the selected Ni_2_O_5_ models are listed in Table 4. We are interested in ambient conditions. For this purpose, the pressure and temperature can be ignored. Therefore, they were set to zero in our calculations.

**Table 4:** DFT + *U* Calculated Energy of Formation, Δ*E*1 (1), and Energy of Reaction, Δ*E*2 (2), for Selected Ni_2_O_5_ Models Calculated for One Formula Unit

oxide	SPGR	Δ*E*1 [kJ mol^–1^]	Δ*E*2 [kJ mol^–1^]
Ni_2_O_3_(O_2_)	*C*2/*c*	–338.4	155.3
Ni_2_O_3_(O_2_)	*Pmnm*	–384.3	109.4
Ni_2_O_3_(O_2_)	*C*2/*m*^*EA*^	–427.6	65.9
NiO_2_NiO(O)_2_	*P1̅*	–272.9	220.9

The energy of formation ([Disp-formula eq1]) gains appreciable negative Δ*E*1 values for all four models (the reaction is exothermic), which shows that they are stable relative to their elemental constituents in their standard states. On the other hand, the reaction 2 is strongly endothermic, indicating that the phases are highly unstable relative to the well-known nickel monoxide, which is the most stable nickel oxide phase. The large Δ*E*2 values, especially those involving Ni_2_O_5_ structures beyond the ground state, suggest that the corresponding structures are unlikely to exist. Thus, these results offer one explanation for why oxygen-rich nickel oxide phases beyond NiO_2_ have not yet been reported.

## 4 Conclusions

The chemical identity of oxygen-rich nickel oxides was investigated employing DFT calculations (PBEsol, PBEsol-D3, PBEsol + *U*, and HSEsol) performed for Ni-substituted transition-metal pentoxides M_2_O_5_, which have the potential to host M^5+^ species, mixed valence, as well as alternative ionic species and exhibit a rich polymorphism. Our results have shown that Ni^5+^ is unlikely to stabilize in the oxide environment and will immediately reduce to more stable cations (Ni^4+^–Ni^2+^) through the formation of molecular oxygen species, while both superoxide and peroxide species are equally likely. Several dynamically stable nickel oxoperoxides and oxosuperoxides phases have been predicted. They represent oxygen-rich variations of the already well-known nickel oxide phases and their heterostructures, including O_2_-decorated rock salt NiO and layered CdI_2_-type NiO_2_ with extra oxygen atoms incorporated within the layers, as well as the van der Waals heterostructure of alternating NiO_2_ and NiO­(O_2_). Our results have also revealed a potentially new NiO_2_ layered form with 5-fold Ni atoms. Furthermore, the *C*2/*c* structure, which is common to all known pentoxides, was found to be dynamically stable also in the case with nickel but in the form of Ni_2_O_3_(O_2_). The calculations employing evolutionary algorithms have predicted the ground-state structure with *C*2/*m* symmetry and confirmed the preference for the formation of oxygen-enriched layered CdI_2_-type phases. All predicted models were found to be stable relative to decomposition to the corresponding elements in their standard states but highly unstable relative to reduction to nickel monoxide, the most stable nickel oxide phase. Thus, these results offer one explanation for why oxygen-rich nickel oxide phases beyond NiO_2_ have not yet been reported.

## Supplementary Material



## Data Availability

The data supporting this article have been included as part of the Electronic Supporting Information.

## Notes

Phonon dispersion curves, crystal orbital Hamilton population (COHP) curves, electronic density of states, electronic localization function maps, relative energies (PBEsol, PBEsol + *U*, PBEsol + D3, HSEsol), crystal structures, integrated crystal orbital bond index (ICOBI) and Löwdin charges calculated for molecular O_2_ species, and crystallographic information files of dynamically stable models (PDF)
