# Supplementary material for: Identity of Oxygen-Rich Nickel Oxides as Oxosuperoxides and Oxoperoxides and Their Heterostructures
Source: Inorg Chem. 2025 Jul 25;64(30):15402–12. doi: 10.1021/acs.inorgchem.5c01477 (PMC12326353; doi:10.1021/acs.inorgchem.5c01477)
Supplement: Supplementary file 1 [file ic5c01477_si_001.pdf]

Supporting information

Electronic Supplementary Information for Identity of oxygen-rich nickel oxides as oxosuperoxides and oxoperoxides and their heterostructures

Radovan Bujdák[a], and Mariana Derzsi[a*], and Kamil Tokár[a,b]

*mariana.derzsi@stuba.sk

[a] - Advanced Technologies Research Institute, Faculty of Materials Science and Technology in Trnava, Slovak University of Technology in Bratislava, 917 24 Trnava, Slovakia
[b] - Institute of Physics, Slovak Academy of Sciences, 845 11 Bratislava, Slovakia

List of Figures

S1: Phonon dispersion curves of Ni_2O_5 models (DFT) . . . S-2
S2: Phonon dispersion curves - $P2_1/m$ (DFT) . . . S-3
S3: COHP analysis of Ni_2O_5 models (DFT) . . . S-4
S4: Electronic Density of states (eDOS) of Ni_2O_5 models (DFT+U) . . . S-5
S5: ELF analysis of for Ni_2O_5 models (DFT) . . . S-6
S6: Relative energies of calculated models (DFT+U) . . . S-7
S7: Visualization of broken $I4/mmm$ structure. . . . S-8
S8: Top view of layers in (a) $C2/m$ and (b) $Pmnm$ structures. . . . S-9
S9: $P1$ structure obtained from the $P2_1/m$ (V_2O_5 type) . . . S-10
S10: Phonon dispersion curves - $P1$ (DFT) . . . S-11
S11: $C2/c$ structure - AFM configurations . . . S-11

List of Tables

1 Integrated Crystal Orbital Bond Index (ICOBI) and Löwdin charge calculated with DFT-PBEsol for the molecular $(O_2)^{1-}$ and $(O_2)^{2-}$ species of the four dynamically stable Ni_2O_5 models. . . . S-12
2 Relative energies calculated for the four dynamically stable Ni_2O_5 with different DFT methods [kJmol^{-1}]. Energies are calculated per formula unit. . . . S-12
3 Crystallographic information for DFT-calculated $C2$/c Ni_2O_5 structure. . . . S-12
4 Crystallographic information for DFT-calculated P-1 Ni_2O_5 structure. . . . S-13
5 Crystallographic information for DFT-calculated $C2/m$ Ni_2O_5 structure (EA ground state structure). . . . S-13
6 Crystallographic information for DFT-calculated $Pmnm$ Ni_2O_5 structure. . . . S-14
7 Relative energies of ferromagnetic (FM), antiferromagnetic (AFM1, AFM2) and non-magnetic (NM) configurations of $C2/c$ model calculated by PBEsol+U method. Energies are calculated per formula unit. . . . S-14

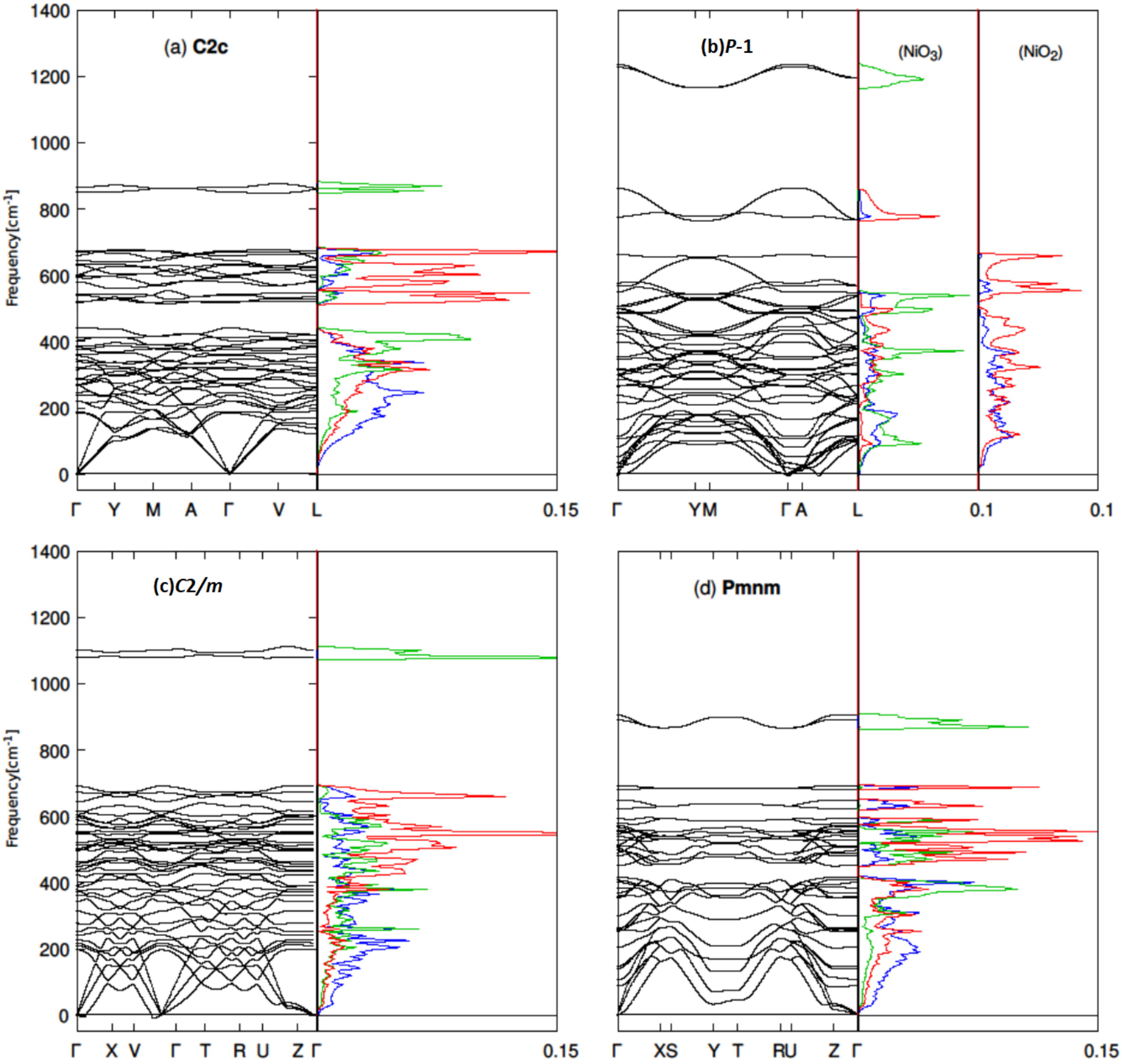

Figure S1: DFT-calculated phonon dispersion curves and projected DOS of selected Ni_2O_5 models: (a) - $C2/c$, (b) - $P1$, (c) - $C2/m$ EA ground-state and (d) - $Pmnm$. Projected DOS for $P1$ model is split into two figures to present different layers in the model separately. Legend: Ni - blue, O-O species - green, O species - red.

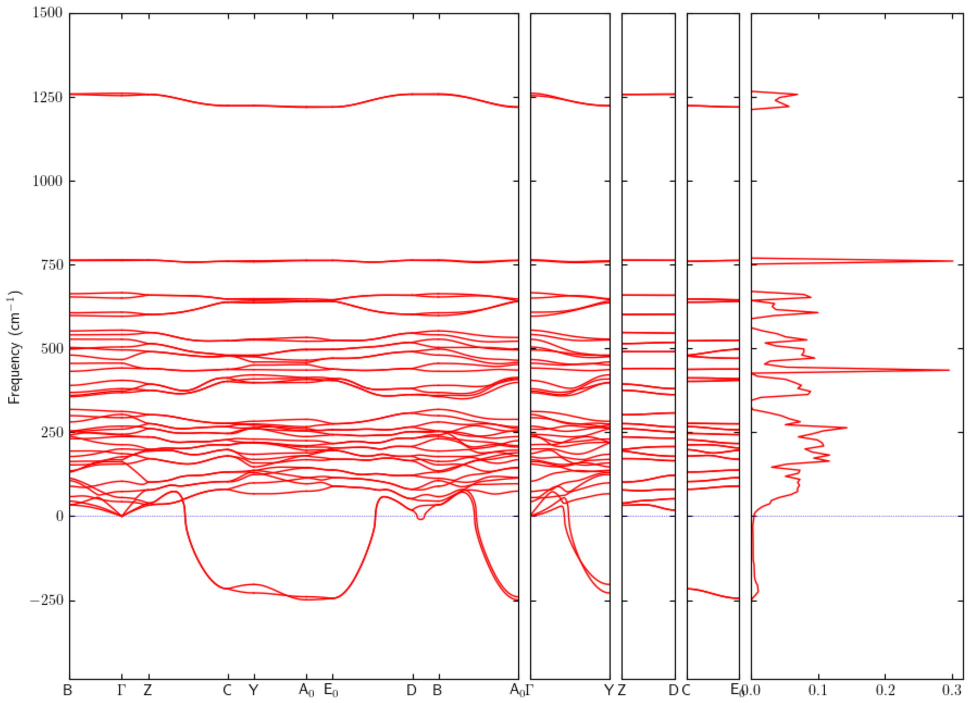

Figure S2: Phonon dispersion curves calculated with DFT for the layered $P2_1/m$ Ni_2O_5 structure, which was obtained from the $Pmmn$ (V_2O_5 type) after symmetry lowering.

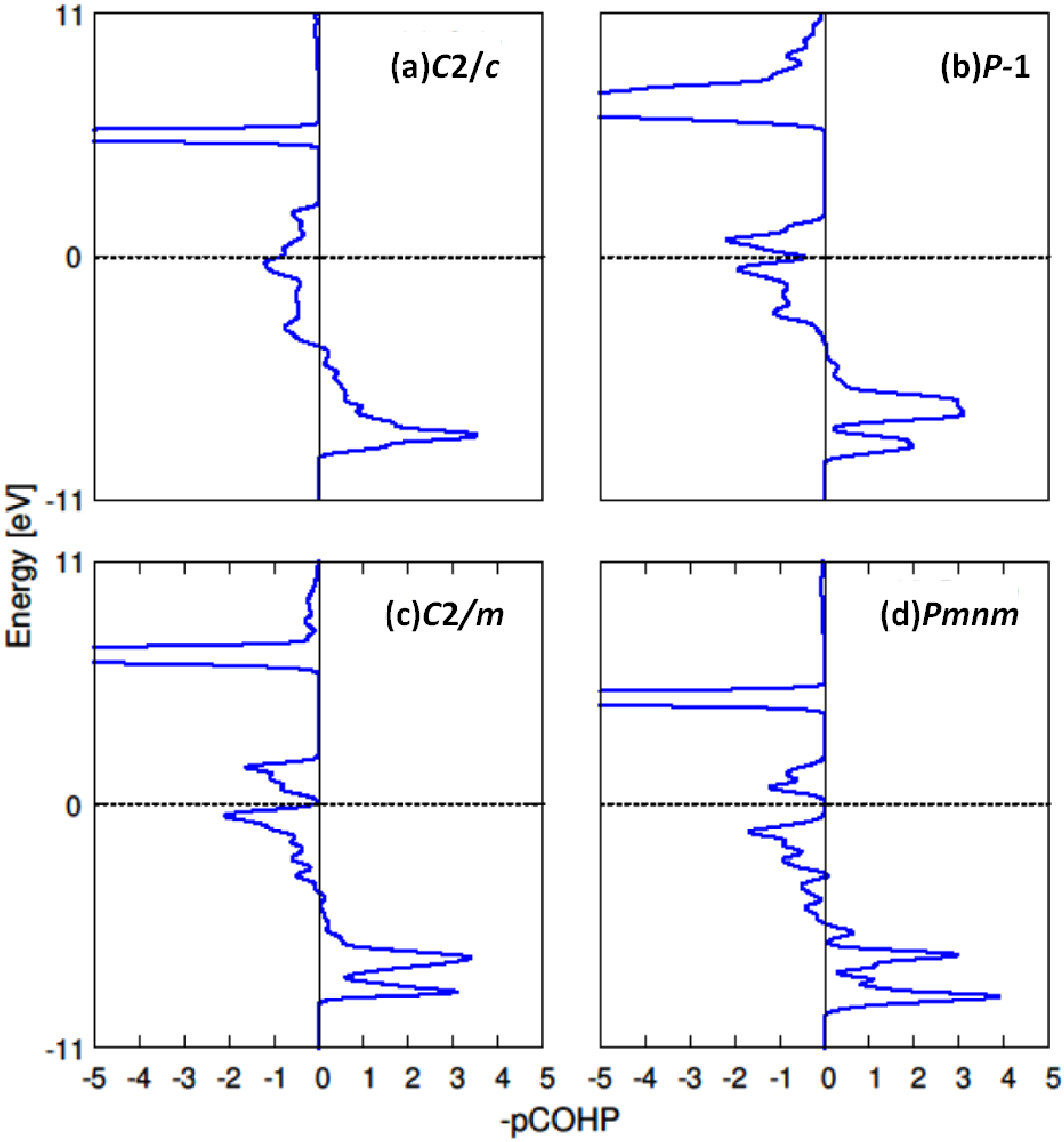

Figure S3: COHP analysis of bonding situation within O_2 dimers calculated with DFT for four dynamically stable Ni_2O_5 models: $C2/c$ (a), $P{-}1$ (b), $C2/m$ (c) and $Pmnm$ (d).

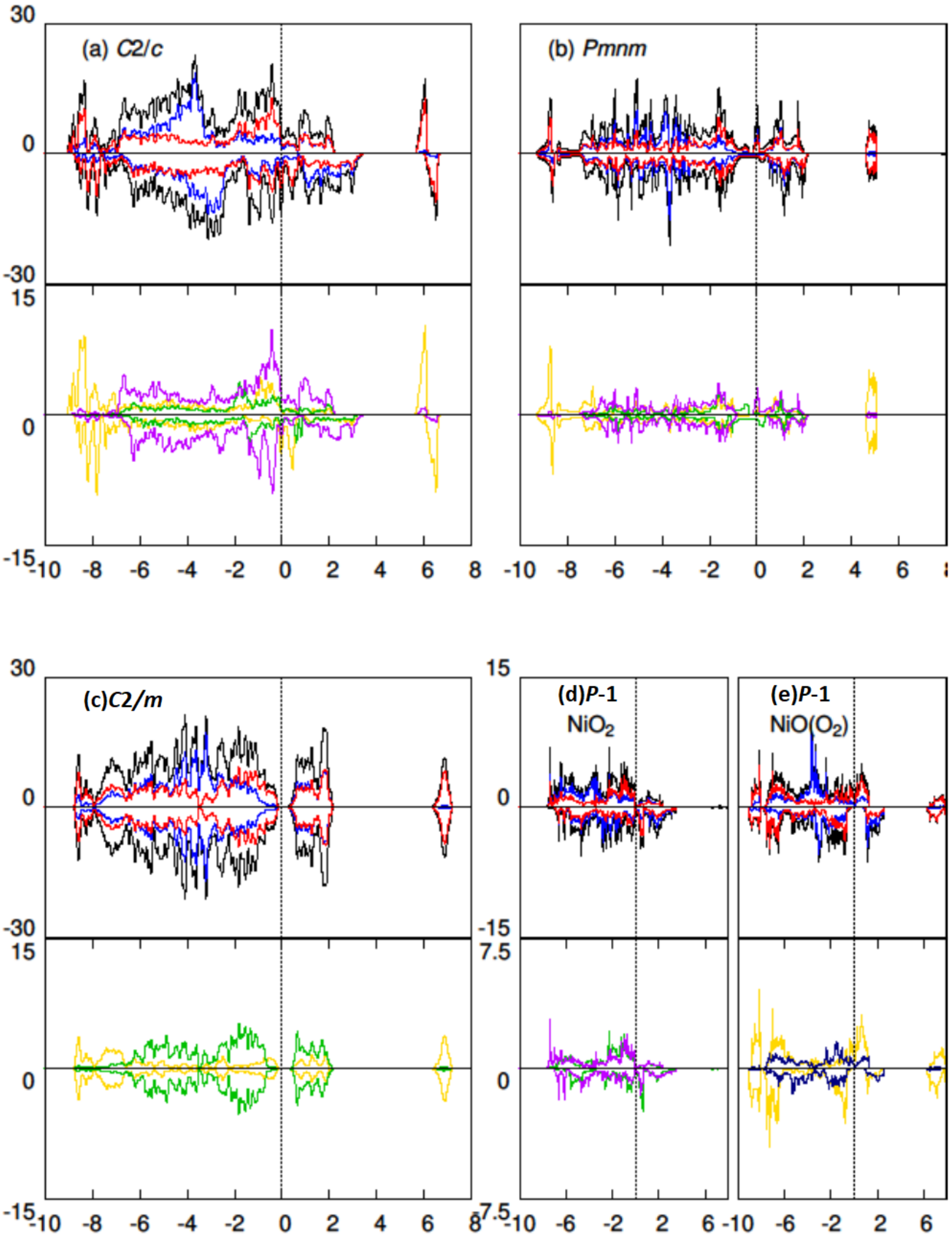

Figure S4: Electronic density of states (eDOS) calculated with DFT+U for four dynamically stable Ni_2O_5 models: $C2/c$ (a), $Pmnm$ (b), $C2/m$ (c) and $P{-}1$ (d,e). In each panel, the top plot contains total Ni and O projected contributions. In the bottom plot, the O eDOS is split into contributions from molecular O_2 species and 2- to 4-coordinated O atoms. Legend: total DOS - black, Ni - blue, O - red, molecular O_2 - yellow, 2-fold O - green, 3-fold O - purple, 4-fold - dark blue.

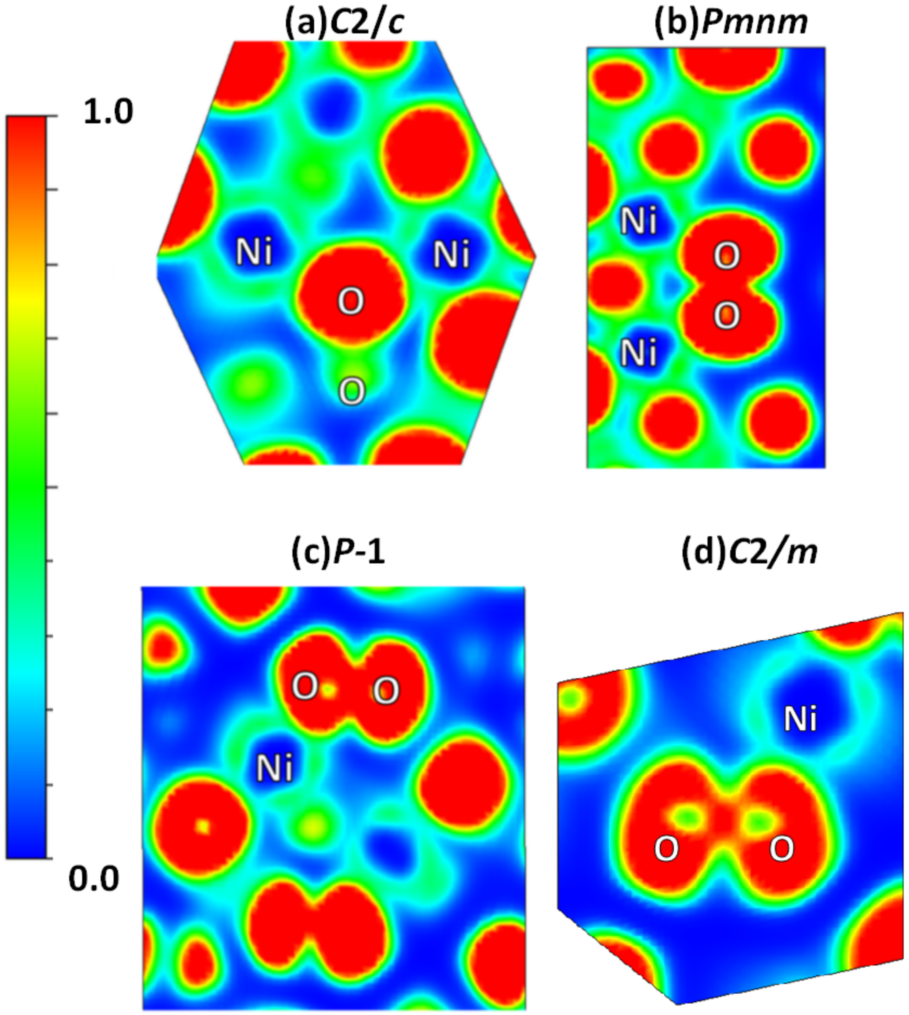

Figure S5: Sections of electron localization function analysis for the four dynamically stable Ni_2O_5 models (DFT): $C2/c$ (a), $Pmnm$ (b), $P-1$ (c) and $C2/m$ (d). Sections run directly through Ni-O bonds, where O atoms are part of O_2.

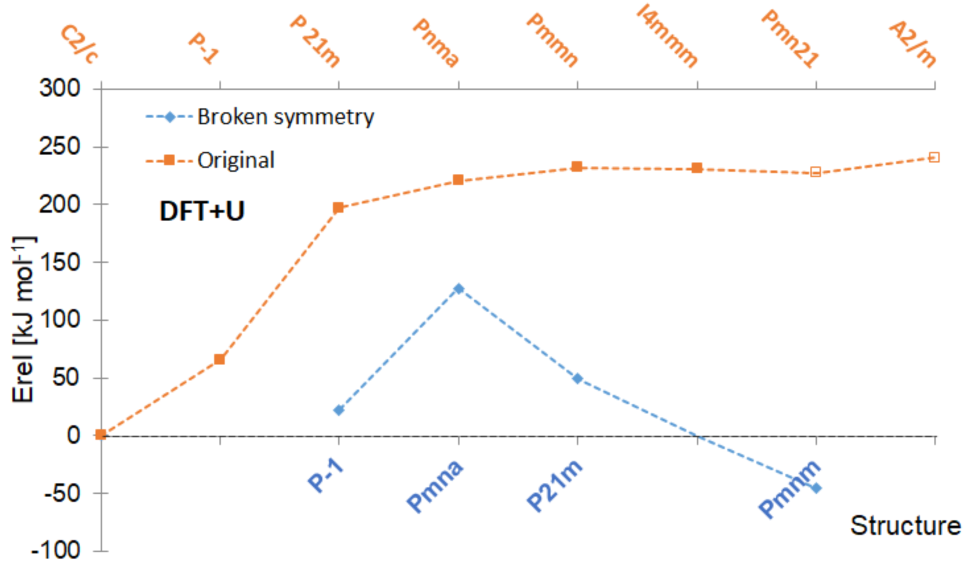

Figure S6: Relative energies of DFT+U calculated Ni_2O_5 models. Orange symbols represent structures constrained by symmetry of original structure models. Blue symbols represent structures with released symmetry constraints. Models were calculated both with and without spin-polarization. Only energetically preferred solutions are visualized - filled symbols represent magnetic solutions and empty symbols represent non-magnetic solutions.

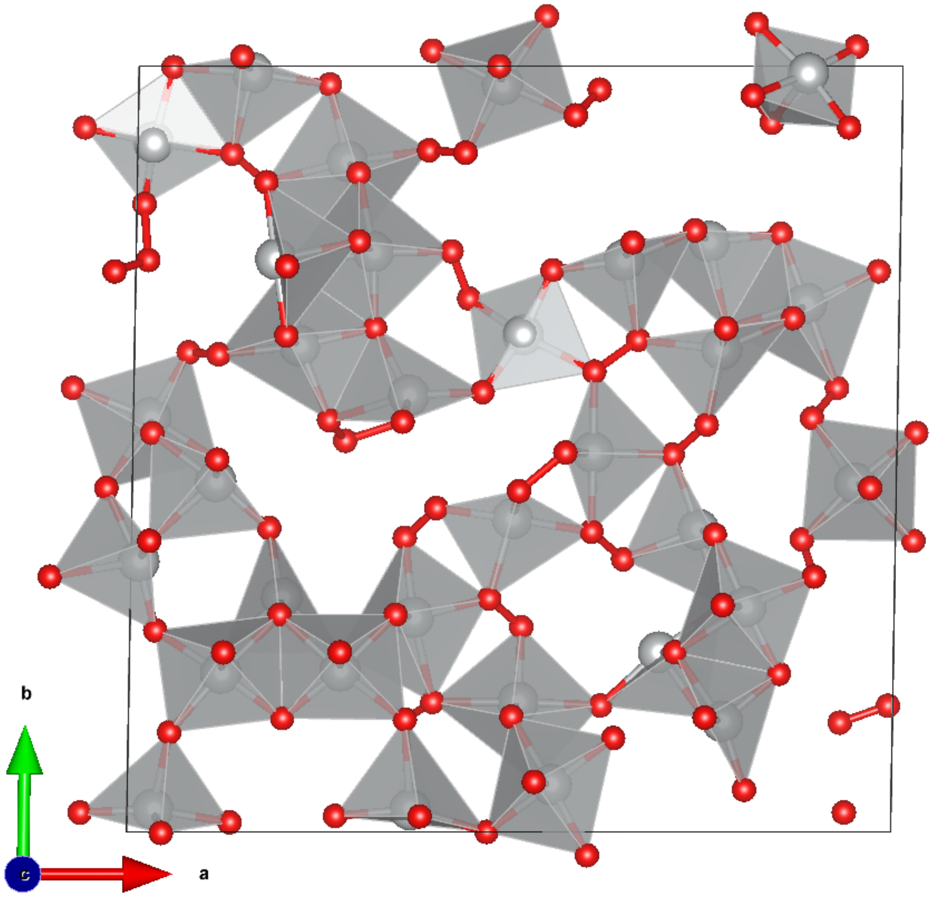

Figure S7: Visualization of $P1$ Ni_2O_5 structure obtained by symmetry lowering from the $I4/mmm$ structure (Nb_2O_5 type). It consists of numerous metal-oxygen coordination polyhedra (3- to 6-fold) without any apparent structural leitmotive.

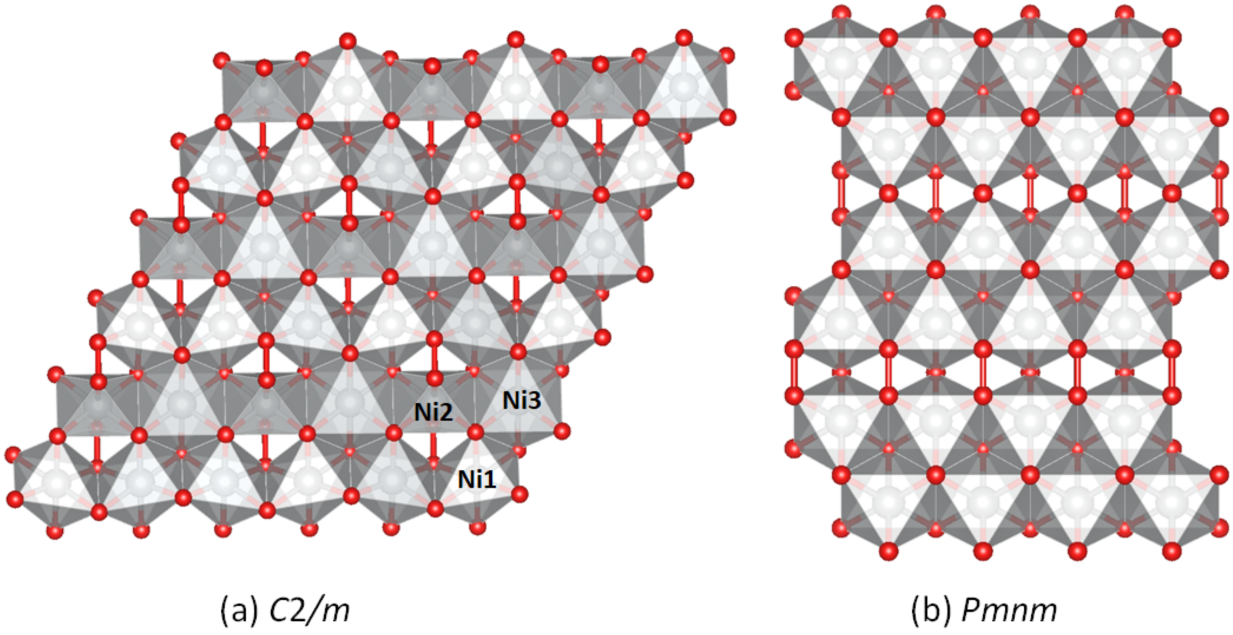

Figure S8: Top view of layers in (a) $C2/m$ (EA ground state) and (b) $Pmnm$ structures.

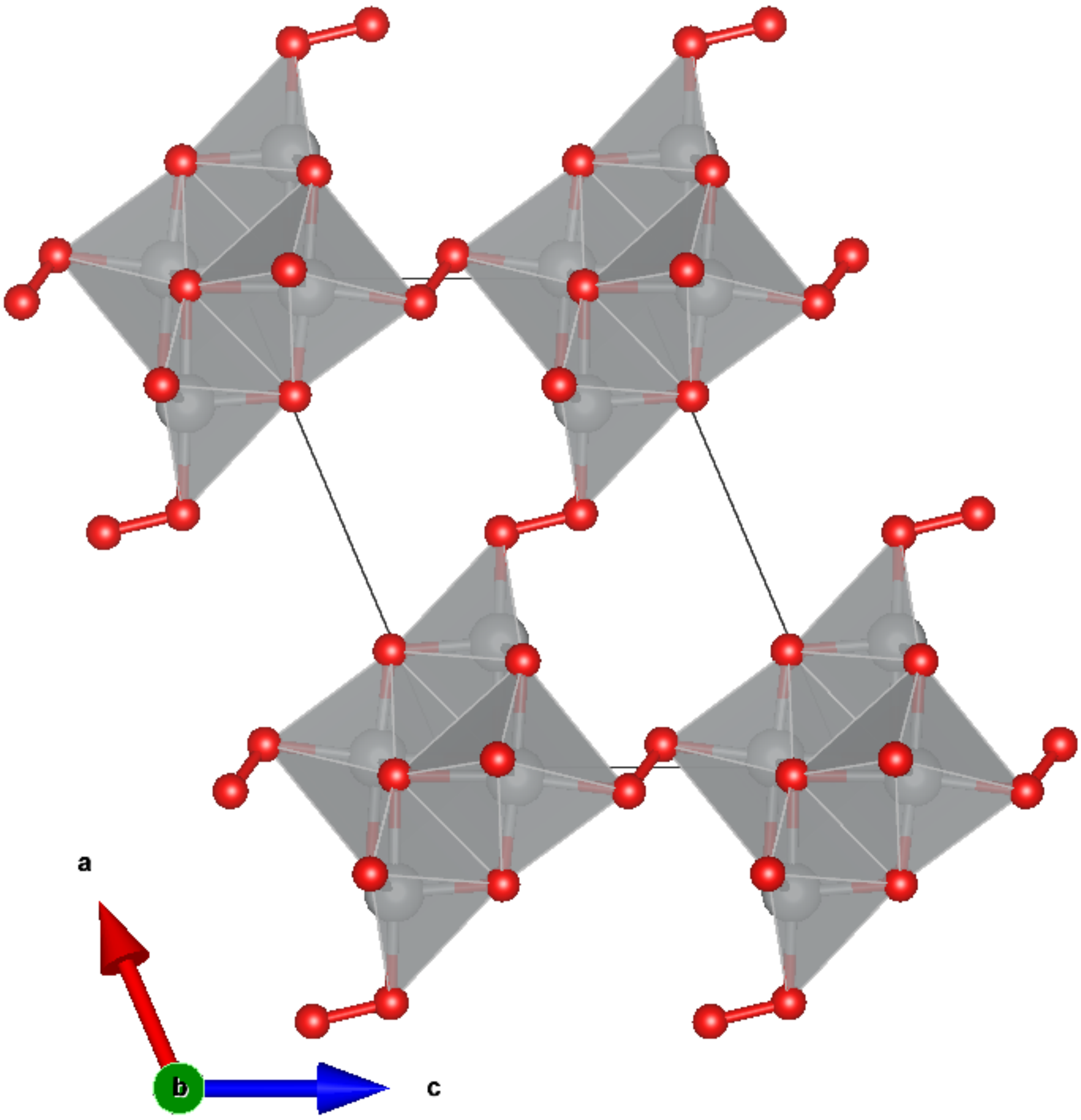

Figure S9: Dynamically unstable $P1$ structure obtained from the $P2_1/m$ (V_2O_5 type) after Ni substitution and removal of symmetry constraints during DFT optimization.

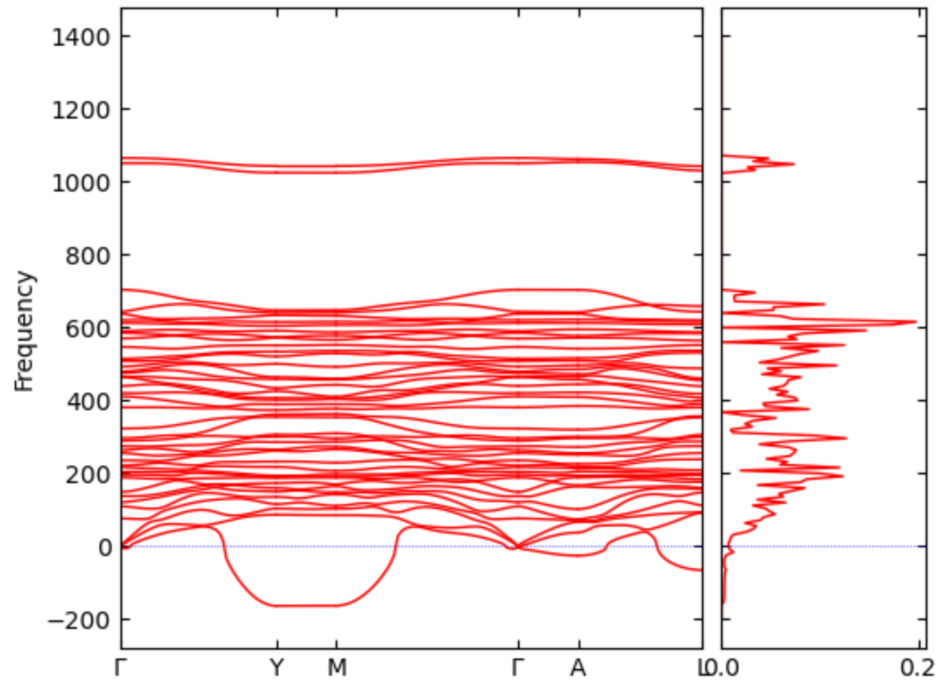

Figure S10: Phonon dispersion curves calculated with DFT for the $P1$ Ni_2O_5 structure, which was obtained from the $P2_1/m$ (V_2O_5 type) by symmetry lowering.

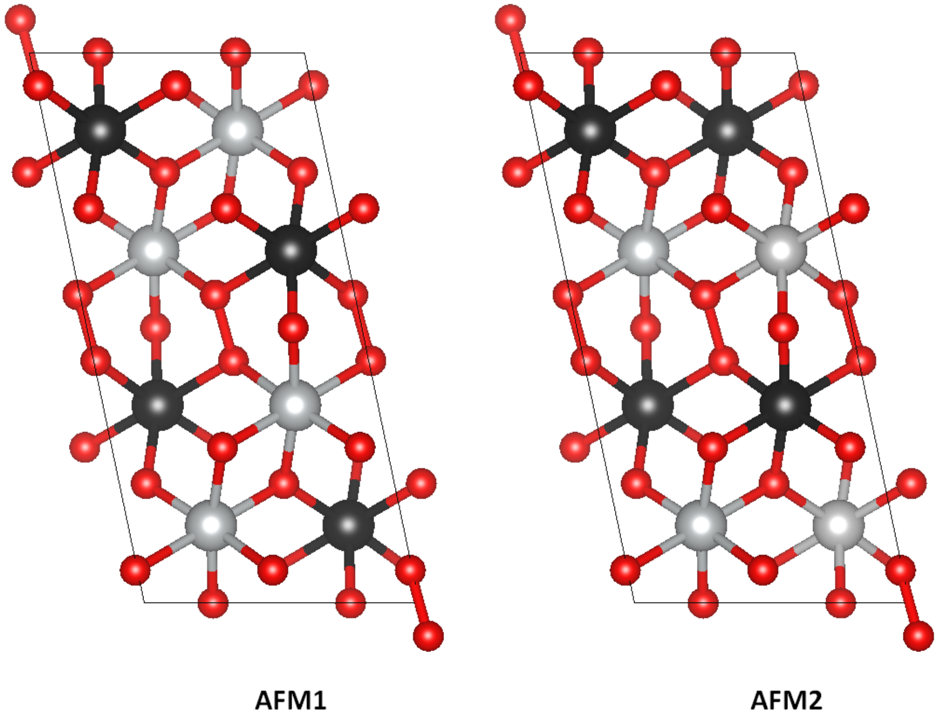

Figure S11: Two AFM configurations of $C2/c$ model considered in energy calculations tests (Table 3, ESI). Black and gray atoms represent spin up/down

Table 1 Integrated Crystal Orbital Bond Index (ICOBI) and Löwdin charge calculated with DFT-PBEsol for the molecular $(O_2)^{1-}$ and $(O_2)^{2-}$ species of the four dynamically stable Ni_2O_5 models.

Structure	Species	ICOBI	Löwdin charge
$C2/c$	$(O_2)^{2-}$	1.04	-0.29
$Pmnm$	$(O_2)^{2-}$	1.01	-0.31
$C2/m$	$(O_2)^{1-}$	1.11	-0.18
$P-1$	$(O_2)^{1-}$	1.21	-0.18

Table 2 Relative energies calculated for the four dynamically stable Ni_2O_5 with different DFT methods [$kJmol^{-1}$]. Energies are calculated per formula unit.

Structure	PBEsol	PBEsol+U	PBEsol+D3	HSEsol
$C2/c$	157.87	177.50	125.13	71.52
$Pmnm$	90.43	86.45	86.08	53.01
$C2/m$	0	0	0	0
$P-1$	284.59	309.17	280.62	115.88

Table 3 Crystallographic information for DFT-calculated $C2$/c Ni_2O_5 structure.

Space group		$C2$/c	
a [Å]		10.768	
b [Å]		4.435	
c [Å]		5.251	
α [°]		90.0	
β [°]		101.4	
γ [°]		90.0	
Fract. coord.:	x	y	z
Ni_1	0.140470	0.264844	0.300155
O_1	0.000000	0.482712	0.250000
O_2	0.059328	0.043249	-0.014427
O_3	0.719023	0.018591	0.092940

Table 4 Crystallographic information for DFT-calculated P-1 Ni_2O_5 structure.

Space group	P-1		
a [Å]	3.449		
b [Å]	3.532		
c [Å]	12.392		
α [°]	91.4		
β [°]	97.5		
γ [°]	92.0		
Fract. coord.:	x	y	z
Ni_1	0.285130	0.227550	0.569775
Ni_2	0.552695	0.762395	0.093245
O_1	0.776610	0.113475	0.319550
O_2	0.233820	0.761315	0.470845
O_3	0.377210	0.741195	-0.059360
O_4	0.567735	0.409850	0.320320
O_5	0.081135	0.760315	0.134540

Table 5 Crystallographic information for DFT-calculated $C2/m$ Ni_2O_5 structure (EA ground state structure).

Space group	$C2/m$		
a [Å]	10.457		
b [Å]	5.608		
c [Å]	6.625		
α [°]	90.0		
β [°]	128.9		
γ [°]	90.0		
Fract. coord.:	x	y	z
Ni1	0.250000	0.250000	0.500000
Ni2	0.000000	0.000000	0.500000
Ni3	0.000000	0.500000	0.500000
O1	0.262501	0.000000	0.323445
O2	0.029212	0.242746	0.347082
O3	0.311092	0.000000	0.742975
O4	0.216575	0.000000	0.811814

Table 6 Crystallographic information for DFT-calculated *Pmnm* Ni_2O_5 structure.

Space group	*Pmnm*		
a [Å]		10.662	
b [Å]		4.852	
c [Å]		2.818	
α [°]		90.0	
β [°]		90.0	
γ [°]		90.0	
Fract. coord.:	x	y	z
Ni_1	0.886080	0.503755	0.250000
O_1	0.314945	0.718610	0.250000
O_2	0.036778	0.306980	0.250000
O_3	0.250000	0.284550	0.750000

Table 7 Relative energies of ferromagnetic (FM), antiferromagnetic (AFM1, AFM2) and non-magnetic (NM) configurations of $C2/c$ model calculated by PBEsol+U method. Energies are calculated per formula unit.

Configuration	E_{rel} [kJmol^{-1}]	E_{rel} [eV]
FM	0	0
AFM1	8.76	0.091
AFM2	8.69	0.090
NM	11.71	0.121
